# Human Cancer Protein-Protein Interaction Network: A Structural Perspective

**DOI:** 10.1371/journal.pcbi.1000601

**Published:** 2009-12-11

**Authors:** Gozde Kar, Attila Gursoy, Ozlem Keskin

**Affiliations:** Center for Computational Biology and Bioinformatics and College of Engineering, Koc University, Rumeli Feneri Yolu, Sariyer Istanbul, Turkey; National Cancer Institute, United States of America and Tel Aviv University, Israel

## Abstract

Protein-protein interaction networks provide a global picture of cellular function and biological processes. Some proteins act as hub proteins, highly connected to others, whereas some others have few interactions. The dysfunction of some interactions causes many diseases, including cancer. Proteins interact through their interfaces. Therefore, studying the interface properties of cancer-related proteins will help explain their role in the interaction networks. Similar or overlapping binding sites should be used repeatedly in single interface hub proteins, making them promiscuous. Alternatively, multi-interface hub proteins make use of several distinct binding sites to bind to different partners. We propose a methodology to integrate protein interfaces into cancer interaction networks (ciSPIN, cancer structural protein interface network). The interactions in the human protein interaction network are replaced by interfaces, coming from either known or predicted complexes. We provide a detailed analysis of cancer related human protein-protein interfaces and the topological properties of the cancer network. The results reveal that cancer-related proteins have smaller, more planar, more charged and less hydrophobic binding sites than non-cancer proteins, which may indicate low affinity and high specificity of the cancer-related interactions. We also classified the genes in ciSPIN according to phenotypes. Within phenotypes, for breast cancer, colorectal cancer and leukemia, interface properties were found to be discriminating from non-cancer interfaces with an accuracy of 71%, 67%, 61%, respectively. In addition, cancer-related proteins tend to interact with their partners through distinct interfaces, corresponding mostly to multi-interface hubs, which comprise 56% of cancer-related proteins, and constituting the nodes with higher essentiality in the network (76%). We illustrate the interface related affinity properties of two cancer-related hub proteins: Erbb3, a multi interface, and Raf1, a single interface hub. The results reveal that affinity of interactions of the multi-interface hub tends to be higher than that of the single-interface hub. These findings might be important in obtaining new targets in cancer as well as finding the details of specific binding regions of putative cancer drug candidates.

## Author Summary

Protein-protein interaction networks provide a global picture of cellular function and biological processes. The dysfunction of some interactions causes many diseases, including cancer. Proteins interact through their interfaces. Therefore, studying the interface properties of cancer-related proteins will help explain their role in the interaction networks. The structural details of interfaces are immensely useful in efforts to answer some fundamental questions such as: (i) what features of cancer-related protein interfaces make them act as hubs; (ii) how hub protein interfaces can interact with tens of other proteins with varying affinities; and (iii) which interactions can occur simultaneously and which are mutually exclusive. Addressing these questions, we propose a method to characterize interactions in a human protein-protein interaction network using three-dimensional protein structures and interfaces. Protein interface analysis shows that the strength and specificity of the interactions of hub proteins and cancer proteins are different than the interactions of non-hub and non-cancer proteins, respectively. In addition, distinguishing overlapping from non-overlapping interfaces, we illustrate how a fourth dimension, that of the sequence of processes, is integrated into the network with case studies. We believe that such an approach should be useful in structural systems biology.

## Introduction

Protein–protein interaction networks provide valuable information in the understanding of cellular function and biological processes. With the tremendous increase in human protein interaction data, network approach is used to understand molecular mechanisms of disease [1] particularly to analyze cancer phenomenon. To date, attempts at providing insights into distinct topological features of cancer genes [2]–[5] have illustrated how to improve cancer classification [6],[7] and identified cancer-related subnetworks [8]. Thus, abstract network representation, where proteins are nodes and interactions are edges, is useful for the comprehension of biological processes and protein function in a global sense. However, to characterize interactions with respect to their physical and chemical properties and in particular, to understand *how* a function is exerted, it is essential to include structural details in the networks; such details come from three dimensional protein structures and from protein interfaces. Proteins interact with each other through binding sites [9]–[13]. Interface characteristics are important in determining the specificity and strength of interactions. For example, conserved modes are used to distinguish biological from crystal interactions [14]. Different in residue composition, transient and obligate complexes have different strength of interactions; the former mostly rely on salt bridges and hydrogen bonds whereas for the latter, hydrophobic forces are more dominant [15],[16]. In terms of geometrical concern, if two proteins interact through a large interface with high complementarity, they will probably interact with high specificity and high affinity [17]. Physical interactions through interface residues also determine whether the binding will be promiscuous or specific.

Structural knowledge of proteins is also critical in identifying whether a binding site is specific or multiply used. Since each protein has almost a fixed surface area, it can have a limited number of binding sites. How can a hub protein interact with tens of other proteins through its binding sites? This question implies that whereas some binding sites are distinct, others should be used to bind to several different proteins. Therefore, the same or overlapping binding sites should be frequently and repeatedly used in hub proteins making them promiscuous [18]. With this in mind, Kim et al. [19] distinguished overlapping from non-overlapping interfaces in their structural interaction network to determine interaction behavior. They classified network hubs into single-interface and multi-interface. The former have at most two distinct binding interfaces and the interactions exclude each other whereas the latter have more than two binding interfaces with most of the interactions being possible simultaneously.

Knowing that cancer-related proteins are more likely to act as hubs [2] in protein interaction networks, the questions that arise are what features of cancer-related proteins make them act as hubs and how is it possible for them to bind to many different proteins with varying affinity. To address these questions, as distinct from previous structural studies [19]–[25], here we integrate protein-protein interfaces into a structural network, focus on cancer-related proteins and investigate the interface properties of cancer/noncancer protein interactions in order to shed light on the details of interaction. We provide a detailed analysis and comparison of six interaction networks: 1) the human protein-protein interaction network, (PIN), 2) the human cancer-related protein-protein interaction network, cPIN, a sub-network of the first. Then, we characterize the interactions in these networks by combining three-dimensional protein structures. Thus, we have: 3) the network constructed by selecting genes for which three-dimensional protein data is available, SPIN, a sub-network of the first, 4) the human cancer-related structural protein-protein interaction network, cSPIN, a sub-network of SPIN. We map the known structural data into these networks whenever a complex structure is available. For the rest, we predict the complex structures of the interactions through structural templates and hot spots using PRISM [26],[27]. The last two resulting networks are “structural interface” networks: 5) human structural protein interface network (iSPIN) and 6) structural cancer-related protein interface network (ciSPIN). These six networks are analyzed and compared to highlight the advantages of using structures. Our results reveal that cancer-related proteins tend to interact with their partners through distinct interfaces, corresponding mostly to multi-interface hubs and constituting the nodes with higher essentiality in the network. In addition, they have smaller, more planar and more hydrophilic binding sites compared to those seen in non-cancer proteins which may indicate low affinity and high specificity of the cancer-related interactions.

## Results/Discussion

### Structural protein interface network (iSPIN)

**Figure 1:**
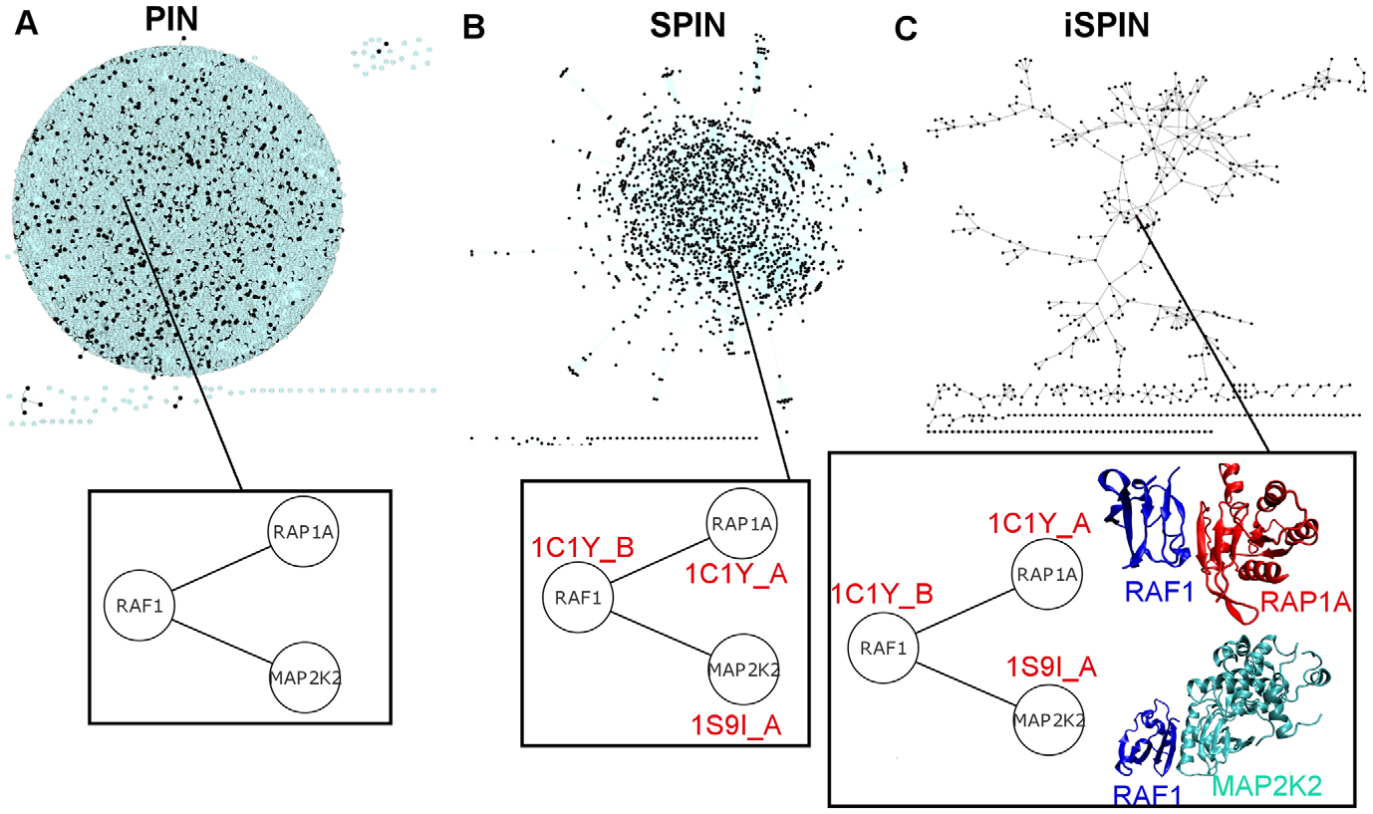
Representation of PIN,SPIN and iSPIN. In A) proteins in PIN are represented; the ones colored black have PDB IDs and the ones colored blue do not have PDB IDs. In B) The proteins with PDB ID and interactions among them constitutes SPIN. In C) The proteins with PDB ID and protein interface information and their interactions constitutes iSPIN. The zoomed representations give idea about what type of information each network contains; PIN is an abstract representation of interactions, SPIN is a subset of PIN with information of PDB IDs, and iSPIN contains the most detailed information including protein interfaces into network. All the networks are visualized using Cytoscape [76].

We illustrate how to obtain a structure-integrated network from PIN: The seed network is the human protein-protein interaction network (PIN) where the nodes are proteins and the edges are interactions. We determined which proteins in this network have structural information in Protein Data Bank (PDB) [28] and constructed a subnetwork with the extracted structures called SPIN (see Methods for the details). To further integrate protein interfaces into SPIN, we mapped the known structural data of complexes into SPIN whenever a complex structure was available. If a known structure was not available for an interaction, we predicted the complex structures of the two interacting proteins using structural templates and hot spots through PRISM [26],[27]. The resulting network, which includes known complexes in PDB and predicted complexes (from PRISM) contains interface knowledge and is called iSPIN. The subsets of PIN, SPIN and iSPIN, which contain cancer-related interactions, are called cPIN, cSPIN and ciSPIN, respectively (See Methods section for further information). Table 1 lists the number of proteins and interactions in each network. In Table 1, “known complex in PDB” column represents the number of interactions for which three dimensional protein structures are available in PDB. The three networks (PIN, SPIN, iSPIN) are illustrated in Figure 1.We should note that there was a dramatic decrease in the number of proteins when going from PIN to SPIN. As seen in Figure 1, while PIN contains information about gene interactions, SPIN only contains those with PDB IDs. And finally iSPIN contains the information at the residue level; protein interfaces. Although we provide a topological analysis of the networks, the main concern of this study is to present interface analysis of cancer-related proteins and, in addition, to predict which interactions can and cannot occur simultaneously and ultimately, to emphasize the importance of using structures in network studies.

**Table 1:** The number of proteins and interactions in each network.

Network name	Protein	Interaction	Known complex in PDB
**PIN**	13584	85083	206
**cPIN**	8990	27413	149
**SPIN**	1702	5312	206
**cSPIN**	1303	3221	149
**iSPIN**	534	549	206
**ciSPIN**	381	363	149

### Analysis of interface properties in iSPIN

We present the interface properties of interactions such as the accessible surface area (ASA), planarity, gap volume index (see definitions below) and residue composition at the interfaces in iSPIN (both predicted and known PDB interfaces). To analyze the properties of interfaces, we used PROTORP [29] (see Methods). First, the analysis of the interface properties throughout the whole network (iSPIN) is presented. Next, the analysis is restricted to subsets of genes having common phenotype, molecular function or biological process.

### Cancer proteins have smaller, more planar, less tightly packed and less hydrophobic binding sites compared to non-cancer proteins

Physical properties of interfaces were computed for the interactions in iSPIN. We classified the interactions into two groups: “cancer-related interactions” are those in which at least one partner in a binary interaction is a cancer-related protein and “noncancer interactions” are those in which none of the proteins are known to be involved in cancer. According to these designations, there were 363 cancer-related and 186 non-cancer interactions. Change in ASA (ΔASA) is the difference between the total ASA of monomers and that of the complex. Cancer proteins on average were observed to have smaller ΔASAs (1009.1 Å^2^) than that of noncancer proteins (1242.9 Å^2^) (standard deviations and p-values are summarized in Table 2). Next, we calculated the interface ASA as the sum of ASAs of each interface residue in the complex state. When the interface ASA of the complex structures is considered, it was found that ASA of cancer proteins (2210.9 Å^2^) were smaller than that of noncancer proteins (2628.1 Å^2^). These results indicate that the complex interfaces which are formed through the interactions of cancer proteins are less buried, or likewise, the monomeric surfaces of cancer proteins are less exposed. It is known that transient complexes have smaller interface areas [30]. Our results show that cancer proteins use a smaller surface area while interacting and we know that they have many interaction partners [3], thus it may be hypothesized that they are more likely to be involved in transient interactions. Here, we should note that although standard deviations of the two datasets are high in all cases, i.e. the distributions of the data sets are highly disperse, p-values at 5% confidence interval are small indicating the significance of the difference between two means of cancer-related and noncancer interfaces.

**Table 2:** Average interface properties of cancer and non-cancer interactions. ± in brackets refers to standard deviation.

Interface property	Cancer-related interactions	Non-cancer interactions	p-value (at α = 0.05)
ΔASA (Å^2^)	1009.1(±611)	1242.9(±942)	6.2e-005
Interface ASA (Å^2^)	2210.9 (±1475)	2628.1(±1947)	0.0006
Planarity (Å)	2.84(±1.28)	3.06(±1.23)	0.04
Gap Volume Index	2.76(±1.48)	2.54(±1.27)	Not significant (0.07)
% Polar residues in interface	29.7 (±14.8)	30.7 (±13.5)	Not significant (0.14)
% Non-polar residues in interface	27.1 (±13.6)	28.8 (±12.9)	0.007
% Charged residues in interface	43.2 (±16.6)	40.5 (±15.4)	0.006

We also investigated the complementarity of the interfaces. Gap volume provides a measure of complementarity and closeness of packing of the interface between the two interacting proteins by measuring the volume of empty space between them. Gap volume index is the ratio of gap volume to the interface area; it estimates the volume enclosed between any two molecules, delimiting the boundary by defining a maximum allowed distance from both interfaces [17]. For the cancer related interactions, the average gap volume (5076.8 Å^3^) was found to be smaller than the average gap volume of noncancer interactions (5574.5 Å^3^) (p-value = 0.038 at α = 0.05). This is an outcome of the smaller interfaces of the cancer proteins since volume is proportional to the surface area. On the other hand, the average gap volume indices for these two categories were 2.76A° and 2.54 A°, respectively (p-value = 0.07 at α = 0.05). This means cancer related interactions are less optimized in terms of complementarity indicating that, the complementarity and packing of two types (cancer/noncancer) are distinguishable from each other.

Planarity indices are used to analyze the shapes of the interfaces. The planarity of the interface is defined as the rmsd of the interface atoms from the least-squares plane fitted through all interface atoms. The larger the planarity index, the less planar the interface, and, conversely, the smaller the planarity index, the more planar the interface [9]. For cancer-related interactions, the average planarity index (2.84) was smaller than that of non-cancer interactions (3.06) with p-value 0.04 indicating that cancer-related interfaces are more planar. It is known that there is a high correlation between the planarity of the interfaces and their ASAs [18]. As the ASAs of the interfaces increase, the planarity index also increases, and the interfaces become less planar, deviating from their principal axes. It is also known that transient complexes usually have more planar interfaces [30]. Here, consistent with previous findings, we observed that cancer proteins use more planar binding sites in their complexes. The results are summarized in Table 2.

Previously, smaller interfaces were shown to display a reduced hydrophobic effect [31]. Residue compositions of interfaces (polar, non-polar or charged) were analyzed in iSPIN and were normalized by the ASA in the complex structures (see Methods). The results revealed that cancer-related interactions show a reduction in hydrophobicity and an increase in charged interactions, and thus have more hydrophilic interfaces than non-cancer interactions. Although, in general, it is agreed that protein-protein interfaces are highly hydrophobic and hydrophobicity is a dominant force in protein-protein interactions [32], there are also studies indicating the importance of hydrophilic interface regions. Tormo et al. (1999) studied the interactions of NK (natural killer) receptors (which regulates NK cell function) and determined the interface of C-type-lectin-like receptor family (Ly49 A) to be highly hydrophilic and dominated by charged interactions [33]. Charged interactions appear to play important role in our iSPIN interfaces as well, which implies that electrostatics are significant in binding. A recent study indicated that favorable electrostatic interactions were not a prerequisite for stable complex formation between proteins whereas hydrophobic effects were found to be favorable in native complexes [34]. Here, we also observed that cancer related proteins, which are intrinsically more disordered and transient [35], had less hydrophobic interactions than other proteins.

### Hub proteins have smaller, more planar, less tightly packed binding sites than non-hub proteins

We also classified interactions as “hub-involved” or “non-hub-involved”. In hub-involved interactions, at least one protein of the binary interaction is a hub protein, whereas in non-hub-involved interactions, none of the proteins correspond to a hub. There were 455 hub-involved interactions and 94 non-hub-involved interactions. As hub proteins in iSPIN, we considered the hubs of SPIN. We found that, on average, hub proteins tended to form smaller, more planar interfaces with their partners. In contrast to previous studies [36],[37], we found no significant difference in the residue composition of the interfaces (including charged residue content) of hub proteins. In terms of complementarity of the interfaces, hub proteins formed looser complexes (gap volume index of 2.72 versus 2.49). The results are summarized in Table 3. (See first lines in each row)

**Table 3:** Average interface properties of hub and nonhub involved interactions. ± in brackets refers to standard deviation. The first and second lines in p-value column represent the comparison of hub/non-hub and single-interface hub/non-hub interactions, respectively.

Interface property	Hub-involved All hubs Single-interface hubs	Nonhub-involved	p-value (at α = 0.05)
ΔASA (Å^2^)	1011.0 (±434) 1022.1(±374)	1459.9 (±1484)	0.0004 0.003
Interface ASA (Å^2^)	2230.0 (±1326) 2228.1(±1178)	2943.9 (±2691)	0.0030 0.01
Planarity (Å)	2.82 (±1.13) 2.97(±1.13)	3.34 (±1.72)	0.0099 0.18
Gap Volume Index	2.72 (±1.40) 2.53(±1.06)	2.49 (±1.48)	0.0580 0.39
% Polar residues in interface	30.5 (±14.5) 32.5 (±14.6)	29.9 (±13.3)	0.80 0.11
% Non-polar residues in interface	28.0 (±13.3) 28.6(±12.2)	28.1 (±13.1)	0.77 0.59
% Charged residues in interface	41.5 (±16.4) 38.8(±0.16)	42.0 (±15.4)	0.77 0.03

Some hubs are single-interface (communicating with their partners by using the same interface) whereas others are multi-interface. The hub proteins of SPIN with more than two interactions in iSPIN were classified as either multi-interface or single-interface hubs resulting in 79 hub proteins, of which 42 were multi-interface and 37 were single-interface. Interestingly, when we compared the interfaces of these two types of hubs, we observed that they had different compositions. Interfaces of multi-interface hubs were usually similar to non-hub interfaces (data not shown). On the other hand, interfaces of single-interface hubs were more polar and less charged than multi-interface hubs and non-hub proteins (See the second lines in each row of Table 3).

### Gene clustering based on phenotype information, molecular function or biological process

The most populated phenotypes observed among cancer genes in iSPIN are leukemia, breast cancer and colorectal cancer, for which there are 55, 22 and 23 related interactions in iSPIN, respectively. Phenotype information was obtained from OMIM [38] which is a compendium of human genes and genetic phenotypes. We compared the interface properties of these cancer related interactions with the same number of interfaces of non-cancer interactions. For all of the phenotype groups, cancer related interfaces showed a reduction in interface ASA and ΔASA compared to noncancer ones. In addition, cancer related interfaces were more planar and less tightly packed.

If the difference in interface properties is important enough, it would be possible to classify a protein as cancer-related or non-cancer by analyzing its interface. Thus, to check whether the data on interface properties can be assessed for classification purposes, we used Weka [39], a machine learning software for data analysis. The training sets included equal number of cancer-related and non-cancer interfaces. The experiments were performed using 10-fold cross validation with several classifiers using four interface features; interface ASA, ΔASA, planarity and gap volume index. (See Methods for the details of the classification procedure) For example, using support vector machine (SVM) as the classifier algorithm, interfaces were ranked as cancer or noncancer related with an accuracy of 61%, 71% and 67% for leukemia, breast cancer and colorectal cancer, respectively. The relatively poorer accuracy of leukemia might be the outcome that there are many distinct subgroups of leukemia which we combined all in one here. The results obtained using SVM classifier are summarized in Table 4. The results using all classifiers are given as supplementary information (Text S1).

**Table 4:** Cancer/noncancer classification analysis and statistical test results for iSPIN interface data, iSPIN clustered data according to phenotype, molecular function or biological process. In the first column, cr stands for cancer-related interfaces and ncr stands for noncancer interfaces. The second column gives the classification performances; first line is accuracy and second line is weighted precision value. The third column lists features (mean values, standard deviations) used in classification for cancer and noncancer interfaces. The last column is the significance of mean values and standard deviations.

Group	Accuracy Precision	Cancer/Noncancer Interface ASA ΔASA Planarity Gap V.I.	p-value (at α = 0.05)
Phenotype: Leukemia (55 cr – 55 ncr)	0.61 0.64	1863.3 (±1207.9)/2425.8 (±1207.9) 850.86 (±346.91)/1147.5 (±633.31) 2.482 (±0.762)/2.939 (±1.390) 2.862 (±1.285)/2.423 (±0.985)	0.0007 0.0287 0.4128 0.1125
Phenotype: Breast cancer (22 cr – 22 ncr)	0.71 0.77	1908.5 (±623.82)/2672.2 (±1257.3) 822.87 (±290.40)/1343.6 (±670.89) 2.361 (±0.601)/3.269 (±1.455) 2.306 (±1.138)/2.239 (±0.9478)	0.03 0.0047 0.1079 0.9159
Phenotype: Colorectal cancer (23 cr – 23 ncr)	0.67 0.73	1923.8 (±533.87)/2790.4 (±1352.8) 978.07 (±325.92)/1428.5 (±771.66) 2.781 (±1.003)/3.472 (±1.724) 2.547 (±1.748)/2.229 (±0.9272)	0.0167 0.04 0.2917 0.6211
Molecular function: Signal Transducer Activity (65 cr – 65 ncr)	0.53 0.55	2226.3 (±1370.7)/2454.7 (±1726.1) 989.50 (±423.20)/1033.5 (±451.70) 2.886 (±1.347)/2.805 (±1.141) 2.764 (±1.178)/2.612 (±1.201)	0.3814 0.62820.85590.3801
Molecular function: Catalytic Activity (84 cr – 84 ncr)	0.58 0.62	2042.8 (±823.69)/2758.1 (±2233.0) 963.32 (±340.09)/1277.1 (±627.45) 2.916 (±1.446)/3.171 (±1.320) 2.496 (±1.066)/2.577 (±1.232)	0.0085 0.0006 0.1078 0.6185
Molecular function: Nucleic Acid Binding (31 cr – 31 ncr)	0.58 0.60	2229.9 (±1504.6)/2567.5 (±870.98) 913.30 (±382.07)/1308.0 (±508.19) 2.717 (±1.458)/3.217 (±1.193) 2.240 (±0.959)/1.934 (±1.031)	0.0188 0.0016 0.0324 0.1471
Molecular function: Transcription Regulator Activity (23 cr – 23 ncr)	0.63 0.64	2668.6 (±2031.3)/2877.9 (±1250.0) 1106.0 (±548.00)/1504.1 (±587.72) 2.615 (±0.897)/3.600 (±1.486) 2.442 (±1.091)/2.011 (±1.111)	0.1803 0.0224 0.0182 0.1732
All data in iSPIN (363 cr – 186 ncr)	0.66 0.44	2210.9 (±1476.0)/2628.1 (±1947.5) 1009.1 (±611.77)/1243.0 (±942.69) 2.843 (±1.285)/3.056 (±1.233) 2.760 (±1.482)/2.543 (±1.275)	0.0006 6.2e-005 0.0429 0.0798
iSPIN equal # of instances (186 cr -186 ncr)	0.54 0.54	2199.6 (±1436.5)/2628.1 (±1947.5) 1029.7 (±732.01)/1243.0 (±942.69) 2.954 (±1.492)/3.056 (±1.233) 2.820 (±1.651)/2.543 (±1.275)	0.0058 0.0013 0.1937 0.1088
iSPIN PDB – PDB interfaces (55 cr – 55 ncr)	0.56 0.56	1917.2 (±884.04)/2471.3 (±1240.9) 911.41 (±489.56)/1258.8 (±740.15) 2.723 (±1.316)/3.298 (±1.424) 3.018 (±1.833)/2.278 (±1.149)	0.03 0.0089 0.0228 0.0214
iSPIN predicted interfaces (131 cr – 131 ncr)	0.57 0.58	2186.6 (±1146.4)/2694.0 (±2177.8) 989.81 (±347.54)/1236.3 (±1018.3) 2.727 (±0.880)/2.955 (±1.135) 2.584 (±1.020)/2.654 (±1.312)	0.0051 0.0035 0.2427 0.7967

We also classified the genes in iSPIN according to the molecular function and biological process of each protein obtained from the Gene Ontology slim terms [40]. Among the most common molecular functions were signal transducer activity, catalytic activity, nucleic acid binding and transcription regulator activity. Interfaces were classified as cancer related with an accuracy of 53%, 58%, 58% and 63% for signal transducer activity, catalytic acitivity, nucleic acid binding and transcription regulator activity, respectively. For the last three molecular functions, interface properties showed noticeable differences for cancer and noncancer interactions. However, for signal transducer activity function (65 cancer related-65 noncancer interfaces), the interface properties were quite similar. We observed that cancer/noncancer interfaces can be distinguished to a greater extent when the genes are classified according to common phenotype rather than molecular function. For the common phenotype case, in our interface datasets, only cancer genes share the phenotype and noncancer genes would have different phenotype properties. On the other hand, for molecular function case, all genes share the same molecular function irrespective of being cancer/noncancer. The relatively poor classification performance by using molecular functions indicates that functionally related proteins might have similar interface characteristics regardless of being cancer-related. Similarly, no discriminative characteristics between cancer-related and noncancer interface datasets were observed when the proteins were classified according to the biological processes.

The last four rows of the Table 4 shows the results of classification performances without grouping genes according to their phenotypes or functions. When we used all the data in iSPIN (with an unbalanced training set), the performance is poorer than the clustered cases. However, when a more appropriate method (adaboost instead of SVM) was used, comparable performances were obtained (Text S1).

### Topological properties of the networks and relationship with essentiality

**Figure 2:**
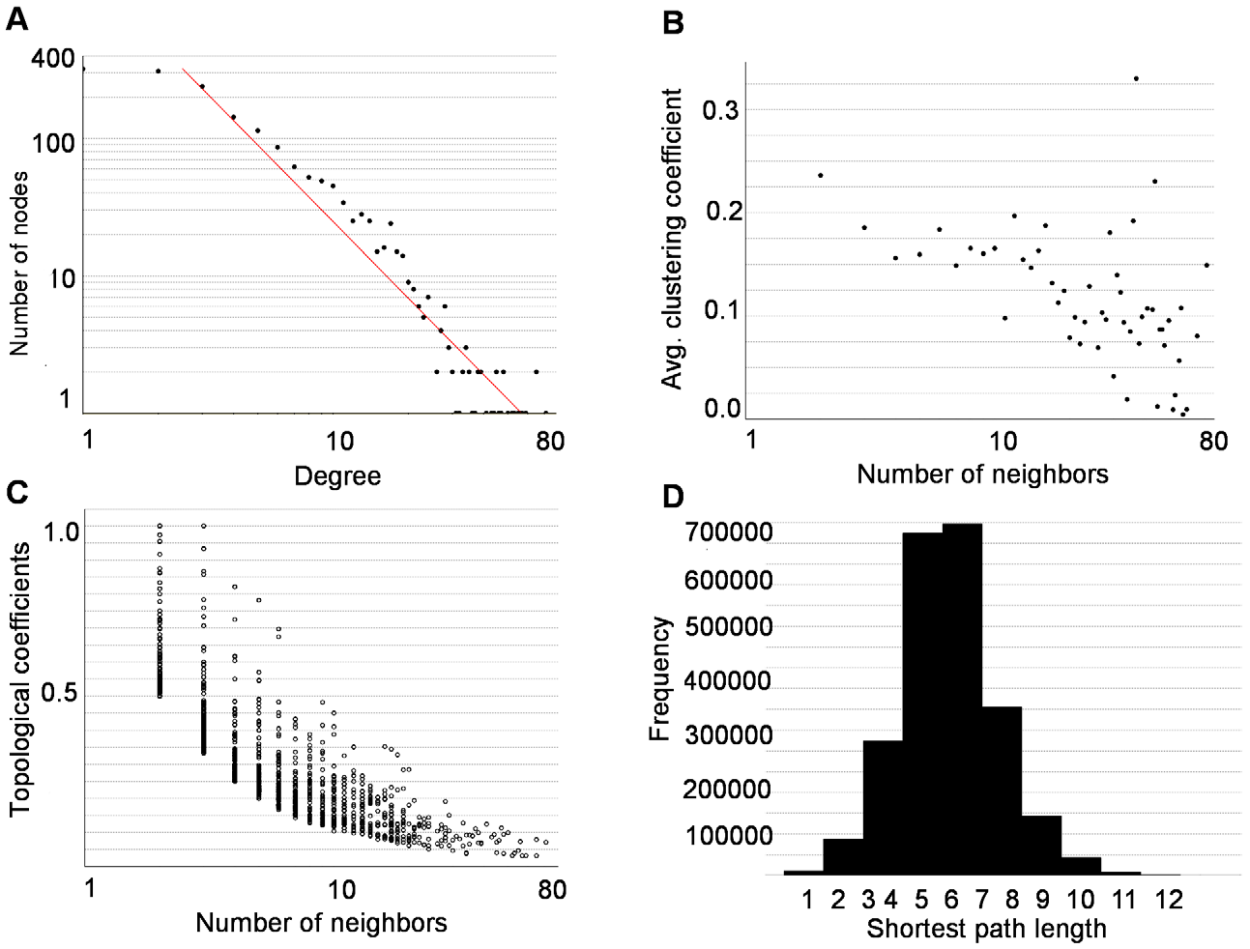
Topological properties of SPIN. (A) Degree distribution of proteins, R^2^ = 0.914 for power law fit (B) Average clustering coefficient (C) Topological coefficients (D) Shortest path length distribution.

Topological properties of protein-protein interaction networks are shown to be useful to characterize proteins functionally [41] and to understand molecular mechanisms of diseases [3],[4]. To address the topological properties of each of our network, we calculated the degree distribution of proteins, which is a measure of the number of proteins' interaction partners. In Figure 2, the topological properties are visualized for SPIN and listed in Table 5. For each network, the degree distribution of the proteins decreases following a power-law (P(k) ∼k^γ^ where k is the number of partner proteins). This implies that the networks have scale-free properties [42]. The average number of neighbors is the average degree of a node in the network. On average, proteins in SPIN have 6.24 interaction partners. A normalized version of average degree is the network density showing how densely the network is populated with edges. When structure information was integrated, network density increased. This might indicate that less connected nodes in PIN might be absent in PDB (Table 5). In Figure 2B, the average clustering coefficient, which is a measure of proteins to form clusters in the network [42] is shown. The average clustering coefficient decreases as the number of protein interactions increases, since sparsely connected proteins are neighbors of highly connected proteins (hub proteins). For the hub proteins, the number of neighbors increased, however, the number of connected pairs did not increase as much as the number of neighbors which caused the average clustering coefficient to decrease. This behavior indicates a hierarchical organization in the protein interaction network [42]. In Figure 2B, we see an exception for this case, although some nodes are highly connected, their average clustering coefficients are also high (>0.30) (upper right corner of the figure). This indicates the occurrence of dense subnetworks, in which hubs mostly interact with other hub proteins. (Such subnetworks in SPIN are explained and visualized in the next section) In Figure 2C, the topological coefficient which is a relative measure for the extent to which a protein shares neighbors with other proteins, [43] is displayed. The decreasing behavior of the topological coefficient as the number of interactions of a protein increases confirms the modular network organization; neighbors of hub proteins are not more connected than the neighbors of sparsely connected proteins. Figure 2D shows the shortest path length distribution and indicates that proteins are closely linked. The topological properties of other networks (PIN, cPIN, cSPIN, iSPIN, ciSPIN) showed similar trends to those of SPIN explained above. When cancer related networks were compared with the whole networks (cPIN with PIN, cSPIN with SPIN and ciSPIN with iSPIN), the average clustering coefficient values were lower; i.e., the proteins have a lower tendency to form clusters. This is reasonable since cancer proteins are the key nodes that link different pathways and they spread throughout the network to function in these pathways. For example, the Cancer Cell Map (http://cancer.cellmap.org/cellmap/), which is a collection of human-focused cellular pathways implicated in cancer, contains ten pathways each having around 100–400 interactions and cancer genes usually function in more than one pathway. Another parameter related to shortest path length is network diameter, which is the largest shortest path length between two nodes providing information about the accessibility of the nodes. The network parameters calculated for each network are displayed in Table 5.

**Table 5:** Network parameters calculated for each network.

Parameter	Network type					
	PIN	cPIN	SPIN	cSPIN	iSPIN	ciSPIN
Number of nodes	13584	8990	1702	1303	534	381
Number of edges	85083	27413	5312	3221	549	363
Clustering coefficient	0.109	0.080	0.143	0.113	0.089	0.051
Characteristic path length	4.086	4.589	4.661	5.064	9.533	8.221
Network diameter	11	11	11	11	23	20
Network density	0.001	0.001	0.004	0.004	0.004	0.005
Avg. number of neighbors	11.27	5.45	6.24	4.94	2.11	1.97

### Topological role & functional distribution of cancer and hub proteins in SPIN and PIN

**Figure 3:**
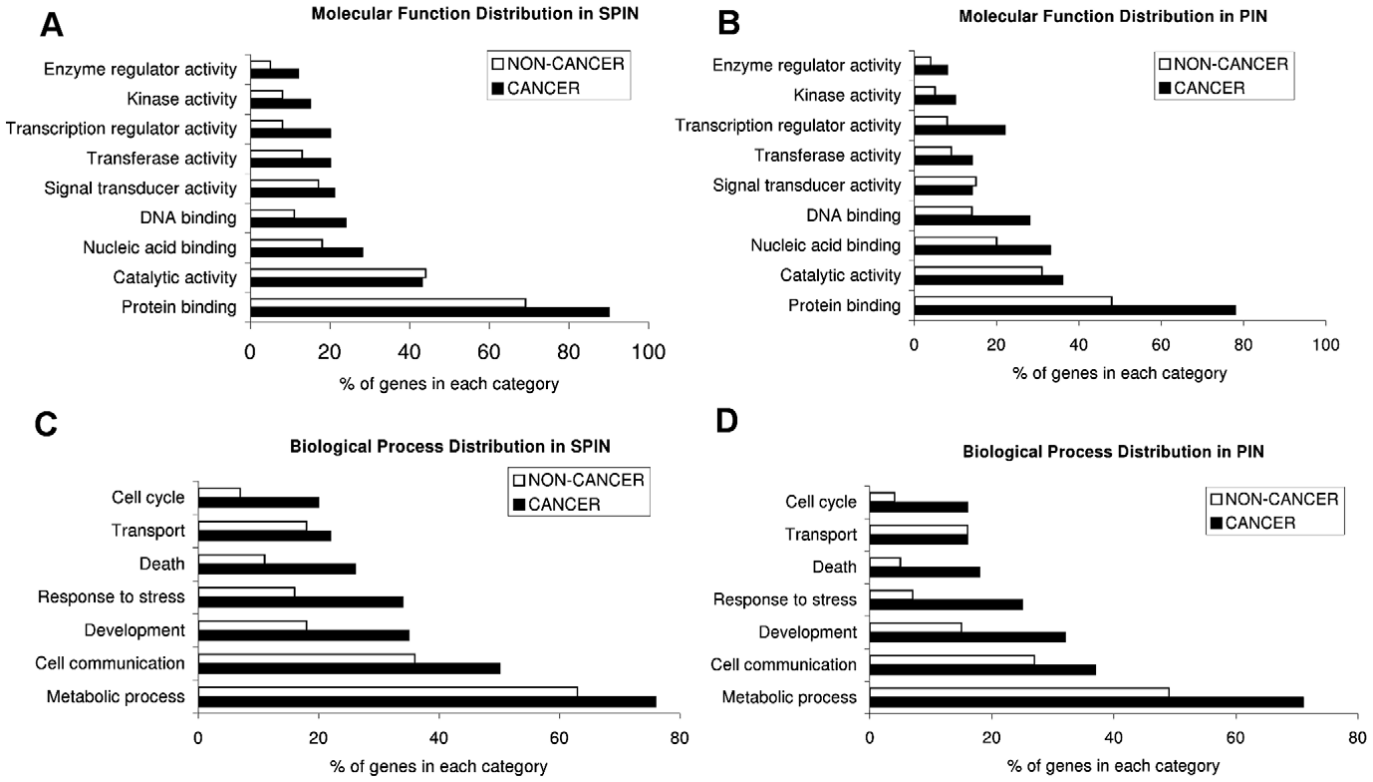
Molecular function and biological process distribution of cancer & non-cancer genes. (A) Molecular distribution of genes in SPIN (B) Molecular distribution of genes in PIN (C) Biological process distribution in SPIN (D) Biological process distribution in PIN.

**Figure 4:**
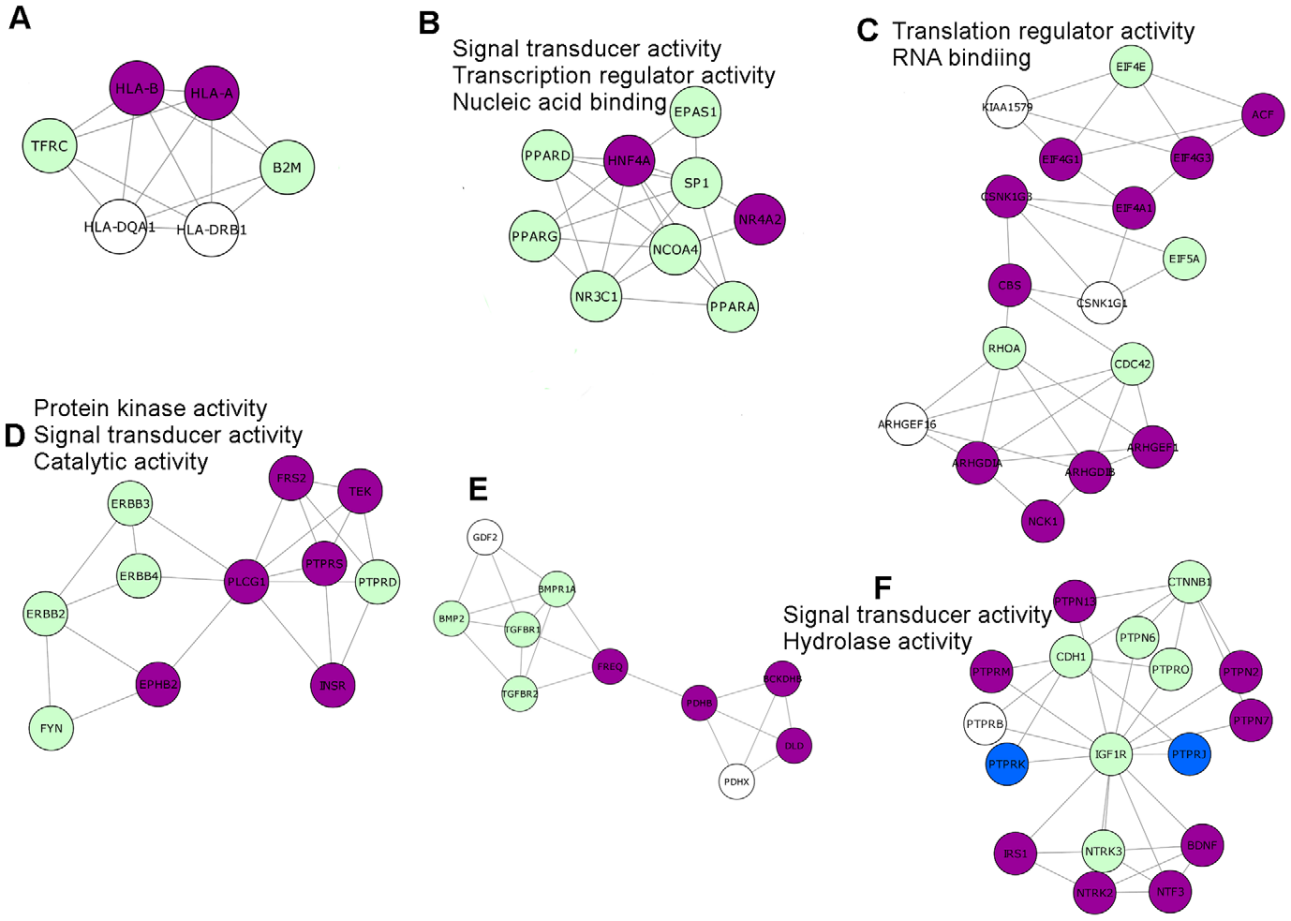
Sub-networks in SPIN. SPIN is clustered into sub-networks, proteins are classified into four categories; cancer-hub, noncancer-hub, cancer-nonhub, noncancer-nonhub are displayed in purple, green, blue and white color, respectively. Over-represented molecular functions (if any) are shown for each sub-network.

Functionally related proteins are more connected than randomly chosen protein pairs [43]. Here, we analyzed the distributions of molecular function of cancer and noncancer proteins and biological process in which they are involved (shown in Figure 3). The results show that in PIN and SPIN, cancer proteins and hub proteins are over-represented in protein binding, signal transducer activity, kinase activity and transcription regulator activity. Previously, Jonsson et al [3] performed a cluster analysis of the human interactome (the so-called ‘PIN’ in this study). They observed that cancer proteins, on average, belonged to more highly populated clusters compared to non-cancer proteins and were involved in multiple cellular processes. Here, we performed a clustering analysis of SPIN using MCODE [44] and obtained subnetworks (see Methods). The first six subnetworks, which were ranked as top six, are shown in Figure 4 (proteins are colored according to four categories; cancer-hub, noncancer-hub, cancer-nonhub, noncancer-nonhub and shown in purple, green, blue and white color, respectively). These subnetworks were compared to SPIN to check if some molecular functions and biological processes were over/under-represented. We observed a common molecular function; signal transduction activity, which is over-represented in three of the subnetworks (subnetworks 2, 4 and 6). In terms of topological properties, these subnetworks showed similarity in the way that they contain hub proteins; subnetworks 2 and 4 contain only hub proteins (cancer or noncancer) and in subnetwork 6; 14 nodes out of 17 are hubs. Thus, we wondered if hub proteins prefer to interact with other hub proteins. Maslov and Sneppen [45] argued that hub proteins do not tend to interact with other hub proteins, but rather prefer to interact with lowly connected proteins. In contrast, Coulomb et al. [46] found that the average degree of nearest neighbors is independent of node degree. We calculated the average degree of hub proteins; we divided the partners of hub proteins into two class; hubs and nonhubs. We found that, on average, hub-nonhub average degree (7.04) was greater than hub-hub average degree (5.06) indicating that hubs do not have a preference to interact with other hub proteins in SPIN. On the other hand, we found that cancer hubs prefer to interact with other hub proteins rather than interacting with non-hubs. Cancerhub – hub average degree and cancerhub – nonhub average degree were 8.49 and 7.16, respectively. The same results are valid for PIN as well. The results support that cancer proteins play central role in the networks and show distinct topological properties than noncancer proteins.

### Hubs are more important than bottlenecks to characterize essential genes

Recently, Yu et al (2007) [47] have analyzed the significance of hubs, proteins with high degree distribution, and bottlenecks, proteins with high betweenness, in the yeast protein-protein interaction network and regulatory networks. They have investigated which quantity, degree distribution or betweenness, is a better predictor of protein essentiality. It was reported that in directed networks, for example in regulatory networks, betweenness is a more important feature in terms of essentiality. In yeast regulatory networks, Yu et al. observed that bottlenecks (both hub-bottlenecks and nonhub-bottlenecks) are generally products of essential genes, whereas hub-nonbottlenecks are not essential at all. When they analyzed the protein-protein interaction network in yeast (undirected network), they found that degree is a much better predictor of essentiality than betweenness since hub-nonbottlenecks are much more essential than nonhub-bottlenecks.

**Figure 5:**
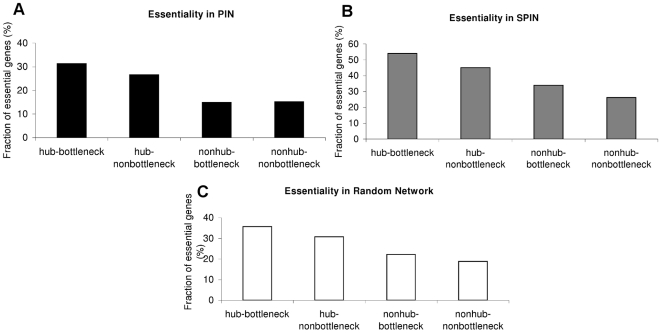
Essentiality of different categories of proteins. A) Essentiality in PIN. B) Essentiality in SPIN. C) Essentiality in random network.

We also investigated how degree and betweenness correlate with essentiality in protein-protein interaction network in human. We classified all proteins into four categories; hub-bottleneck, hub-nonbottleneck, nonhub-bottleneck and nonhub-nonbottleneck. Figure 5 (A, B) show the essentiality of different categories of proteins, in PIN and in SPIN. In addition to these networks, a random network, which is the same size as SPIN and has the same average degree distribution, was generated from PIN. First a protein from PIN was selected randomly. Then, some of the interactions of this protein were randomly selected. The same procedure was applied to the newly selected neighbors until the network size and average degree distribution values were satisfied. As shown in Figure 5, the hub-bottlenecks were found to be the most essential category in all networks. The fraction of essential gene percentages for hub-bottlenecks in SPIN, random network and PIN were 54%, 35% and 31%, respectively. Hub-nonbottlenecks were found to be more essential than nonhub-bottlenecks; i.e. degree is a more important parameter in terms of essentiality in PIN, SPIN and the random network. This finding confirms the hypothesis stated by Yu et al (2007) [47].

Essentiality fractions in SPIN were much higher than the ones in PIN (y-axes of Figure 5A and Figure 5B). The reason for higher fraction of essential genes in SPIN may stem from a possible bias towards well-studied proteins for which structural information is available. Another reason could be a physical bias due to the fact that PIN is a large-scale data. To investigate the reason for this bias, we generated a random network from PIN, which is the same size as SPIN and has the same average degree distribution. Figure 5C displays the fraction of essential genes in this random network. We observed that the fraction of essentiality was higher for the random network than for PIN. However, the values were still much smaller than those for SPIN. Thus, we concluded that the reason for higher essentiality in SPIN probably arose from a bias towards well-studied proteins rather than a physical bias.

### The essentiality of cancer hubs is significantly higher than that of non-cancer hubs

**Figure 6:**
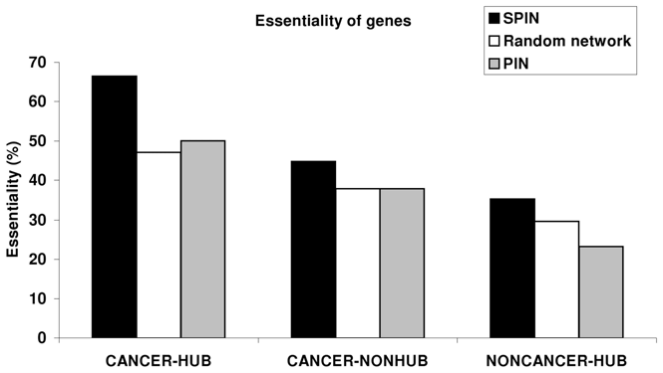
Essentiality of proteins classified as cancer-hub, cancer-nonhub and non-cancer-hub in SPIN, PIN and random network.

Hub proteins are more likely to be encoded by essential genes [48],[49]. In addition, somatic cancer genes are more likely to encode hub proteins [2]. From these, we can hypothesize that essential cancer genes are more likely to encode hub proteins than non-essential cancer genes. Thus, we classified all cancer genes in the networks as hub and non-hub, and observed that cancer-hubs were more essential than cancer-nonhubs, which confirms our above hypothesis; essential cancer genes are more likely to encode hub proteins than non-essential cancer genes. The essentiality percentage in each category, hubs and non-hubs are 50% (total 532) and 37% (total 650) for PIN, 66% (total 158) and 44% (total 286) for SPIN, 47% (total 85) and 37% (total 140) for random network, respectively. The essentiality percentage values are visualized in Figure 6.

Another question is whether cancer or non-cancer hubs are more essential. We found that when we classified the hub proteins as cancer-hubs and non-cancer-hubs, there was a significant difference in essentiality. In SPIN, there were 158 cancer hubs, 66% of which were essential. In contrast, only 28% of the 197 non-cancer hubs were essential. Similarly, in both PIN and the random network cancer hubs were much more essential than non-cancer hub proteins. In PIN the 50% of the 532 cancer hubs were essential, whereas only 24% of the 1801 total non-cancer hub proteins were essential. In the random network, 47% of 85 total cancer hubs were essential, whereas 30% of 246 total non-cancer hub proteins were essential. The fraction of essential genes in cancer hubs and non-cancer hubs for each network are shown in Figure 6. The numbers of essential and nonessential genes are given for each category in PIN, SPIN and random network as supplementary information (Text S1). We should note that essential gene list is obtained on optimal growth/living conditions and if the conditions are changed, for example in case of a disease state such as cancer, a nonessential gene would become essential or vice versa. However, due to the lack of data on essential gene information in cancer cells, we assigned the same set of essential genes to cancer state and non-cancer state. Recently, Luo et. al [50] had an effort to identify the genes essential for growth and related phenotypes in different cancer cells by genetic screening strategy. Since a small fraction of these genes appear in our networks, it is not appropriate to use them in statistical analysis.

### Multi-interface and single-interface proteins: Correspondence with degree, betweenness and essentiality

As discussed above, some hubs are single-interface, that is, they communicate with their partners by using the same interface, whereas others are multi-interface. We investigated to which category, hub-bottleneck or hub-nonbottleneck, multi-interface and single-interface proteins belong. We observed that multi-interface proteins generally corresponded to hub-bottleneck proteins rather than hub-nonbottlenecks (71% of multi-interface proteins are hub-bottlenecks.) When the single-interface proteins were considered, the percentage of hub-bottleneck correspondence decreased to 59%. In other words, 58% of hub-bottleneck proteins were multi-interface and 42% are single-interface. Previously we showed that hub-bottlenecks were the most essential category of proteins in SPIN and in PIN. Here, in the structural interface network, we found that the essentiality of multi-interface hubs (68%) was higher than that of single-interface (52%). This result agrees with a previous finding [19] indicating that the number of interfaces leads to higher essentiality. In addition, Aragues et al. (2007) found that yeast hubs with multiple interacting motifs were more likely to be essential than hubs with one or two interacting motifs [51]. Being more essential and corresponding mostly to hub-bottlenecks, multi-interface hubs are the key points in the protein-protein interaction network.

Cancer proteins in our network are more enriched in multi-interface proteins: 56% of cancer proteins are multi-interface, while 44% being single-interface. This is reasonable since on average, cancer proteins are longer [52] with larger surface areas. To cope with many interactions at the same time, they tend to be multi-interface hubs with distinct interfaces interacting with different proteins. Although cancer proteins tend to have more than one distinct interface, we found that on average their interfaces were smaller, which can indicate that their binding behavior acts similar to that of hub proteins. In addition, the average number of interfaces of cancer multi-interface hubs and noncancer multi-interface hubs were 2.5 and 2.3, respectively. Cancer multi-interface hubs have a greater average number of interfaces. The correspondence of hub-bottlenecks and hub-nonbottlenecks to multi/single interface proteins and the essentiality percentage in cancer/noncancer & multi/single interface proteins are displayed in Table 6.

**Table 6:** Correspondence of HB, H-NB to Multi/single interface proteins and Essentiality % in cancer/noncancer & Multi/single interface proteins HB and H-NB refer to hub-bottlenecks and hub-nonbottlenecks, respectively.

	HB	H-NB	Total
**Multi-interface #**	30	12	42
**Single-interface #**	22	15	37
	**Essentiality percentage (%)**		
**Multi-interface**	68		
**Single-interface**	52		
**Cancer**	76		
**Non-cancer**	42		

### Case Studies

**Figure 7:**
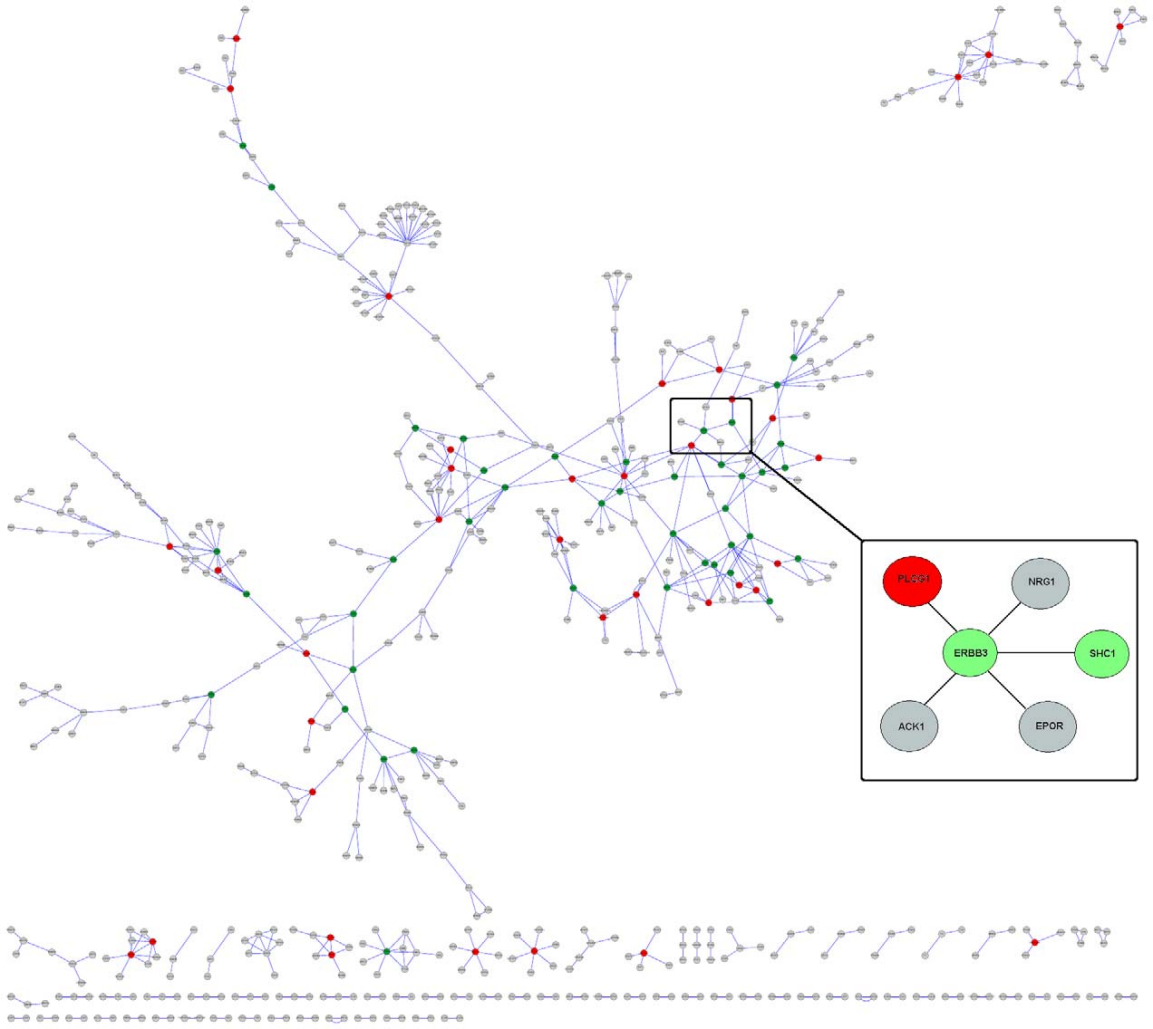
Representation of iSPIN. The nodes colored in green and red are multi-interface hubs and single-interface hubs, respectively. In the zoomed representation, the interactions of a multi-interface hub; ERBB3 is displayed.

The interface information is an asset in predicting which interactions can and cannot co-exist. In other words, it will help to deduce which interactions can occur simultaneously and which are mutually excluded. Addressing this question may add a fourth dimension to interaction maps, that of sequence of processes. Including the *sequence* dimension in structural networks is an immense asset; transforming network node-and-edge maps into cellular processes, and assisting in the comprehension of cellular pathways and their regulation. Here, to characterize the interactions and to infer the order of processes, we present two case studies, first a multi-interface cancer protein and an inhibitor of the protein, and second, a single-interface cancer protein in iSPIN. For the first case study, multi-interface cancer protein, most of the interactions are simultaneously possible whereas for the latter, the interactions are mutually exclusive. In addition to geometrical justification for simultaneous and exclusive interaction behavior, dynamic nature of protein-protein interactions are taken into account. The interacting complexes were refined using FiberDock http://bioinfo3d.cs.tau.ac.il/FiberDock/, which models both side-chain and backbone flexibility. Next, to obtain a quantitative estimation of the importance of the interactions, we used FoldX algorithm [53],[54] for calculating the interaction energy between two proteins, which serves as an estimate for the affinity of the interactions. In Figure 7, a visualization of iSPIN is displayed together with multi-interface and single-interface proteins.

### A multi-interface hub: ErbB3 (Her3)

**Figure 8:**
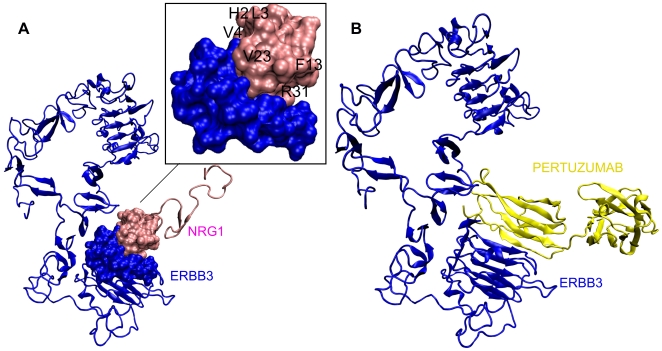
Representation of ERBB3-NRG1 interaction schematically. The interactions are visualised using VMD [78] A) ERBB3 (1m6b_A) and NRG1 (1hae_A) are shown as newcartoon diagram in blue and red color, respectively. The transparent surface represents the interface region. The labeled residues (represented by their Cα atoms) of 1hae_A are reported to be critical for binding in a previous work [58]; i.e. when they are mutated to alanine, the binding affinity for ERBB3 was significantly reduced. B) HER3 (blue) – pertuzumab heavy chain (yellow) is shown. Pertuzumab shares the same interface with NRG1 (see “An inhibitor affecting Erb signaling pathway: pertuzumab” section).

Here we show how the interface information is used to deduce which interactions can and cannot co-exist. If each interaction partner of a hub protein uses a distinct interface on the hub while interacting, then these interactions are more likely to occur simultaneously. In addition, the quaternary structure of the complex should be considered carefully to ensure that the interaction partners do not collide. To demonstrate this idea, we present a so-called ‘multi-interface’ hub protein: ERBB3 (or HER3), which is one of the hub proteins in SPIN. The receptor tyrosine-protein kinase erbB-3 precursor (ERBB3) belongs to EGF receptor subfamily and acts as a heregulin receptor and as an epidermal growth factor receptor. Amplification of this gene and/or overexpression of its protein have been reported in numerous cancers, including prostate, bladder, and breast tumors [55]. According to the KEGG database [56], ERBB3 functions in the ErbB signaling pathway and the Calcium signaling pathway. In the ErbB signaling pathway, NRG1 (neuregulin 1, heregulin), which is a direct ligand for ERBB3, binds and activates ERBB3. We modeled this interaction using the PDB accession codes 1hae_A (NMR structure of heregulin) for NRG1 and 1m6b_A (crystal structure of ERBB3 taken from a homodimer structure) for ERBB3, respectively. PRISM results indicate that these two proteins (1hae_A and 1m6b_A) interact, and using NOXclass [57], we found that the interaction is biologically relevant. After applying flexible refinement by FiberDock, FoldX server [53],[54] was used to calculate the interaction energy (−4.08 kcal/mol). Predicted binding sites on both proteins and interacting residues for NRG1-ERBB3 interaction are shown in Figure 8A. The interaction was experimentally studied in a previous study by Jones et al (1998) [58], where they mutated individual residues of the egf domain of heregulinβ (the same as egf domain of heregulinα-NRG1- except four residues) to alanine in order to determine residues critical for binding receptors and initiating signal transduction. They found that when His^2^, Leu^3^, Val^4^, Phe^13^, Val^15^–Gly^18^, Val^23^, Arg^31^, Lys^35^, Gly^42^–Gln^46^ residues were changed to alanine, binding affinity for ERBB3 was dramatically reduced. We observed that most of these critical residues were included in our predicted binding site for NRG1. In Figure 8A, these residues are labeled.

In the ErbB signaling pathway, NRG1 also binds to ERBB4, and the binding affinity was reported to be similar to that of ERBB3 [58]. According to our interface prediction, ERBB3 and ERBB4 binding interfaces on NRG1 are overlapping; i.e., the same binding site is used for the ERBB3 and ERBB4 interactions. Therefore, NRG1-ERBB3 and NRG1-ERBB4 interactions are mutually exclusive; they cannot occur at the same time.

**Figure 9:**
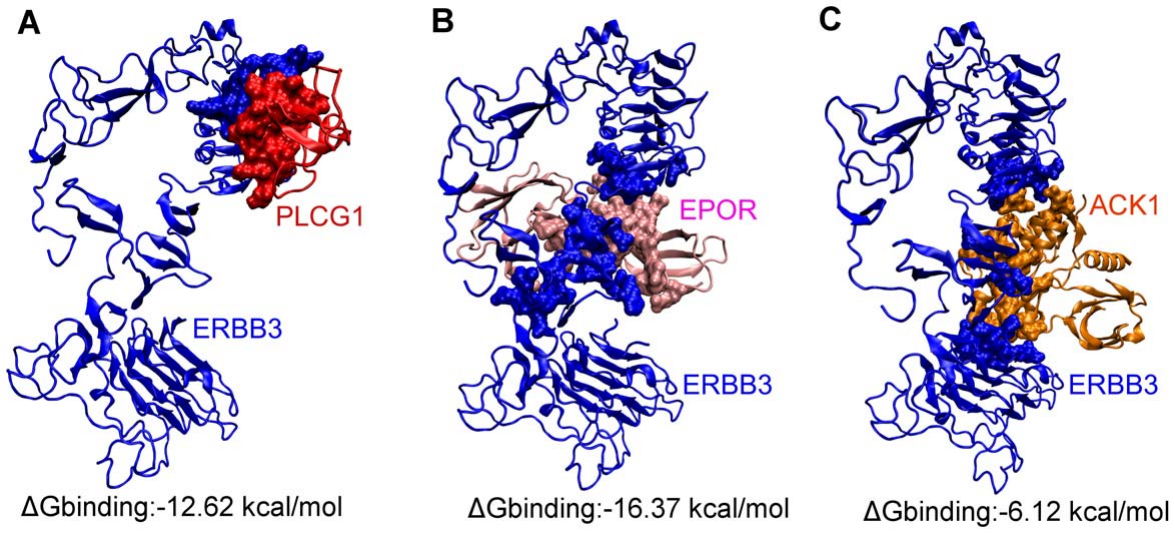
Ribbon diagram and interface representation of ERBB3 interactions with PLCG1, EPOR and ACK1. ERBB3 (1m6b_A), PCLG1 (1hsq_A), EPOR (1eer_B) and ACK1 (1u46_A) are colored in blue, red, pink and orange respectively. Interface residues are shown as spheres. (A) ERBB3-PLCG1 interaction. (B) ERBB3-EPOR interaction. (C) ERBB3-ACK1 interaction.

According to the calcium signaling pathway in KEGG [56], ERBB3 interacts with PLCG1. Although the interaction is not reported in public databases as in DIP [59], BIND [60], in a recent study, it was observed on protein microarrays [61]. PLCG1 (Phospholipase C-gamma-1) is a major substrate for heparin-binding growth factor 1 (acidic fibroblast growth factor)-activated tyrosine kinase. The PDB structure of SH3 domain of PLCG1 is 1hsq. The predicted interface residues of ERBB3-PLCG1 (1m6b_A-1hsq_A) interaction are displayed in Figure 9 labeled as A. The interaction energy between proteins was calculated as −12.62 kcal/mol.

The two other possible interactions of ERBB3 occur with EPOR (Erythropoietin receptor) and ACK1 (Activated CDC42 kinase 1) according to the human interactome constructed by Jonsson and Bates. No experimental confirmation is available for these interactions yet, however, they have high confidence scores to occur in Jonsson and Bates's network [3]. These interactions of ERBB3 were also predicted to interact and further investigated. Subcellular location for ERBB3, EPOR and ACK1 is the cell membrane. EPOR and ERBB3 function as single-pass type I membrane protein. The predicted interfaces for these interactions are illustrated in Figure 9, labeled as B and C.

Our results show that ERBB3 uses at least three different binding sites while interacting. Of these interactions, we propose that ERBB3 cannot interact with EPOR and ACK1 at the same time, because if we model the quaternary structure of ERBB3-EPOR-ACK1 complex, the residues of EPOR and ACK1 will collide. Thus, they cannot bind simultaneously. But, we should keep in mind that proteins are dynamic, and a hinge-like motion of the two domains of ERBB3 can eliminate the collision between EPOR and ACK1.

If we compare their interaction energy, which were calculated as −16.37 kcal/mol and −6.12 kcal/mol for ERBB3-EPOR and ERBB3-ACK1, respectively, ERBB3-EPOR interaction is more favorable. In addition, when ACK1 interacts with ERBB3, it also blocks the interaction of NRG1. In terms of geometrical and energy concern, the simultaneously possible interactions would be ERBB3-PLCG1 (interaction energy: −12.62 kcal/mol) and ERBB3-EPOR, for which the affinity predictions are higher than those of other interactions.

### An inhibitor affecting Erb signaling pathway: pertuzumab

To illustrate the importance of the sequence of processes, we further focused on ERBB3 interactions and investigated how it functions if its partners use the same interface while interacting. In this case the interactions cannot occur at the same time. In general, the HER/erbB family of proteins (EGFR (HER1), HER2, HER3, and HER4) activate intracellular signaling pathways in response to extracellular signals [55]. The signaling mechanism is as follows: first EGFR and HER3 are activated by ligand binding (ligands are EGF and NRG1 for EGFR and HER3, respectively), and then EGFR or HER3 forms heterodimer with HER2 followed by the transphosphorylation of their C-terminal tails. Heterodimer formation of HER2 with EGFR and HER3 induces different pathways. For example, The PI3K/Akt pathway, which is critically important in tumorigenesis, is activated by phosphorylated HER3. The deregulation of signaling functions of the HER family of proteins causes cell transformation and tumorigenic growth [55]. In anti-cancer drug development, EGFR and HER2 proteins are the main targets. For example, pertuzumab, which targets HER2 dimerization region, attempts to inhibit HER2-HER3 or HER2-EGFR interactions.

In a recent study [62] investigating the effect of pertuzumab in lung cancer cells, it was found that pertuzumab blocked NRG1-stimulated phosphorylation of HER3. In contrast, it failed to block epidermal growth factor (EGF)-stimulated phosphorylation of EGFR in human non-small cell lung cancer cell line 11_18. This is somewhat interesting since HER2 uses the same binding region for dimerization with HER3 and EGFR and this region is assumed to be blocked by pertuzumab. However, it may be hypothesized that in addition to its inhibiting effect on dimerization region of HER2, pertuzumab should also affect the ligand binding region of HER3 and EGFR, namely HER3-NRG1 interaction and EGFR1-EGF interaction.

In order to investigate the effect of pertuzumab on HER3-NRG1 interaction, pertuzumab heavy chain (PDB ID 1s78) was docked to HER3 (PDB ID 1m6b). The docked conformation is visualized in Figure 8B. NOXclass results indicate that the docked conformation is biological (biological score is 70%). Although HER2 and HER3 are similar in structure, the interface region on HER2 and HER3 through which the interaction with pertuzumab occurs are not exactly the same in structure, but rather use overlapping regions. We observed that pertuzumab binding interferes with NRG1 binding region, which indicates that pertuzumab may also block ligand binding to HER3 and thus prevent HER3 activation. 36% of interface residues (8 out of 22) of HER3-NRG1 interface are also used by pertuzumab, which makes the interactions of HER3 with NRG1 and pertuzumab mutually exclusive. Both interactions are visualized together and the black surface region shows the shared interface region (see Text S1).

Thus, our results indicate that pertuzumab may block the NRG1 interaction region of HER3. Probably, pertuzumab would not affect the binding of EGF to EGFR and thus it is not effective against (EGF)-stimulated phosphorylation of EGFR in the aforementioned lung cancer cells.

### A single-interface hub: RAF1

If the interaction partners of a hub protein use the same interface region, then these interactions are more likely to be mutually exclusive. For example, in iSPIN, RAF1 has 9 interactions partners which compete for binding. RAF proto-oncogene serine/threonine-protein kinase participates in the transduction of mitogenic signals from the cell membrane to the nucleus and protects cells from apoptosis mediated by STK3. Among its interaction partners, we were able to predict interaction interfaces for CDC25, YWHAZ and MAP2K2, for which interaction energies were calculated as −1.91 kcal/mol, −8.35 kcal/mol, −2.92 kcal/mol, respectively. We should note that all interaction energies were calculated for the comparison of the interactions and the numeric values may not be precise since these are not experimental results.

**Figure 10:**
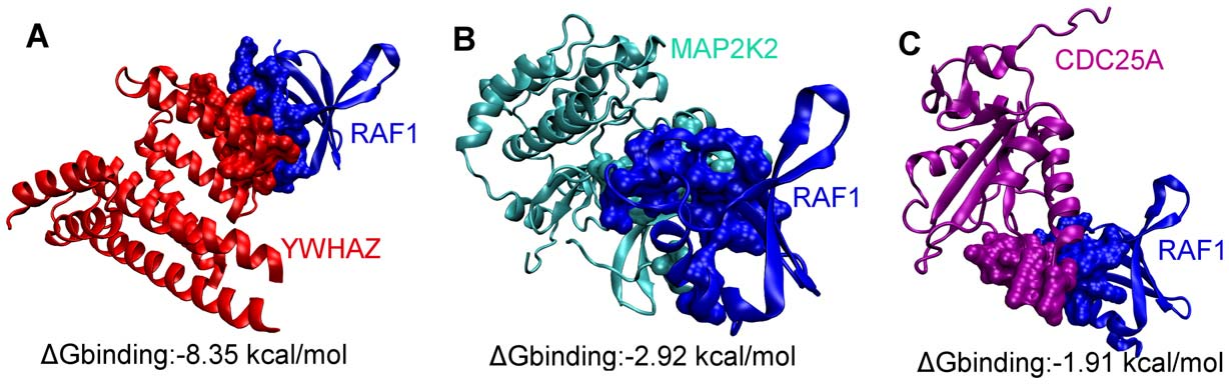
Ribbon diagram and interface representation of RAF1 interactions with YWHAZ, MAP2K2 and CDC25A. RAF1 (1c1y_B), YWHAZ (1qja_A), MAP2K2 (1s9i_A) and CDC25A (1c25_A) are colored in blue, red, cyan and purple respectively. (A) RAF1-YWHAZ interaction. (B) RAF1-MAP2K2 interaction. (C) RAF1-CDC25A interaction. Interaction interfaces of RAF1 through YWHAZ, MAP2K2 and CDC25A are highly overlapping; the interactions are mutually exclusive.

Interaction with RAP1A is a known structure with PDB ID 1c1y. Additional possible interactions of RAF1 in iSPIN are with RALA, DIRAS1, DIRAS2, CCNA2 and RRAD. Although the interface region is not completely the same for each interaction partner, most interface residues are shared (the shared percentage >20, which is the cutoff value for assigning the interface as distinct or same). Thus, these interactions cannot occur at the same time. All three interactions (RAF1-CDC25A, RAF1-MAP2K2, RAF1-YWHAZ) are cancer-cancer related and their affinities are lower compared to ERBB3-EPOR and ERBB3-PLCG1 interactions which are cancer-noncancer related and simultaneously possible. Friedler et al. (2005) [63] observed a highly electrostatic binding site in a cancer protein, p53, interacting with Rad51 and other peptide sequences with different affinity. The results imply that cancer proteins and hubs interact with their partners with high specificity and low affinity. Therefore, it becomes possible for them to bind to many different proteins with varying affinity. Three predicted binding sites are illustrated in Figure 10. In Text S1, RAF1 is displayed with its three binding partners: RAF1 (1c1y_B) is shown in blue, the partners YWHAZ (1qja_A), MAP2K2 (1s9i_A) and CDC25A (1c25_A) are colored in red, cyan and purple respectively. The interface is highly shared which hypothesize that RAF1 is a single-interface protein and involved in mutually exclusive interactions. RAF1 is a protein kinase and a signaling protein; thus, it probably interacts transiently with most of its targets. A recent study confirms this interaction behavior of RAF1, showing that the binding of Cdc25 and of Rad24 (14-3-3 homolog that is important in the DNA damage checkpoint) to Raf-1 is mutually exclusive [64].

### Conclusion

In this work, we analyzed cancer proteins and hub proteins in human protein-protein interaction networks from a structural perspective, and by considering their global behavior in the network.

Integrating three-dimensional protein structures into human protein-protein interaction network revealed important aspects about hubs and cancer-related proteins. Interface property analysis identified the structural tendencies of cancer proteins that assist their binding to multiple proteins. Interfaces of cancer proteins, on average, are smaller in size, more planar, less tightly packed and more hydrophilic than those of non-cancer proteins. Within phenotypes, for breast cancer, colorectal cancer and leukemia, interface properties were found to be discriminating from non-cancer interfaces with an accuracy of 71%, 67%, 61%, respectively.

Hub proteins also have smaller, less tightly packed and more planar interfaces than non-hub proteins. Similar or overlapping binding sites should be used repeatedly in hub proteins, single interface hub proteins, making them promiscuous. Alternatively, multi-interface hub proteins make use of several distinct binding sites to bind to different partners. Interfaces of multi-interface hubs are usually similar to non-hub interfaces. On the other hand, interfaces of single-interface hubs are more polar and less charged than multi-interface hubs and non-hub proteins.

In addition cancer-related proteins tend to interact with their partners through distinct interfaces, corresponding mostly to multi-interface hubs, which comprise 56% of cancer-related proteins, and constituting the nodes with higher essentiality in the network (76%). Cancer proteins are more enriched in multi-interface proteins: 56% of cancer proteins are multi-interface, while 44% being single-interface. This is reasonable since it is known that, on average, cancer proteins are longer with larger surface areas. To cope with many interactions at the same time, they tend to be multi-interface hubs with distinct interfaces interacting with different proteins. Cancer multi-interface hubs have a greater average number of interfaces.

We found that, on average, hub-nonhub average degree (7.04) is greater than hub-hub average degree (5.06) indicating that hubs do not have a preference to interact with other hub proteins in SPIN. On the other hand, we found that cancer hubs prefer to interact with other hub proteins rather than interacting with non-hubs. Cancerhub – hub average degree and cancerhub – nonhub average degree are 8.49 and 7.16, respectively. The same results are valid for PIN as well. The results reveal the well known information that cancer proteins play central role in the networks and show distinct topological properties than noncancer proteins.

Finally, we illustrated, in detail, the interface related affinity properties of two cancer-related hub proteins: Erbb3, a multi interface, and Raf1, a single interface hub. The results revealed that affinity of interactions of the multi-interface hub tend to be higher than that of the single-interface hub. These findings might be important in obtaining new targets in cancer as well as finding the details of specific binding regions of putative cancer drug candidates.

## Methods

### Human protein-protein interaction and cancer-associated protein interaction datasets

We studied the human interactome constructed by Jonsson & Bates (2006) [3] and referred to this network as ‘PIN’. They used an orthology-based method in which BLAST [65] searches were run for the human genome against all proteins in the DIP [59] and MIPS Mammalian Protein-Protein Interaction databases [66]. They analyzed their putative interactions giving confidence scores based on the level of homology to proteins found experimentally to interact and the amount of experimental data available. After ROC curve analysis, with a sensitivity of 85% and specificity of 82%, the human interactome consisted of 108113 binary gene-gene interactions and 13584 genes. From these interactions, the redundant ones, i.e. the interactions for which the RefSeq ID corresponding to the same genes, were omitted. Thereby, the network (PIN) consists of 85083 interactions. The list of cancer genes was taken from the comprehensive census of human cancer genes provided by Futreal et al (2004) [67]. 10724 interactions were cancer-related in this interactome. In addition, we collected a set of known cancer genes from the Memorial Sloan Kettering computational biology website CancerGenes (http://cbio.mskcc.org/CancerGenes/Select.action) using the queries of “tumor suppressor”, “oncogene” and “stability” genes. We combined that list with the known cancer genes of Futreal et al. [67]. Thus, cancer related interactions number increased to 27413.

### Mapping interactions to known 3D structures

**Figure 11:**
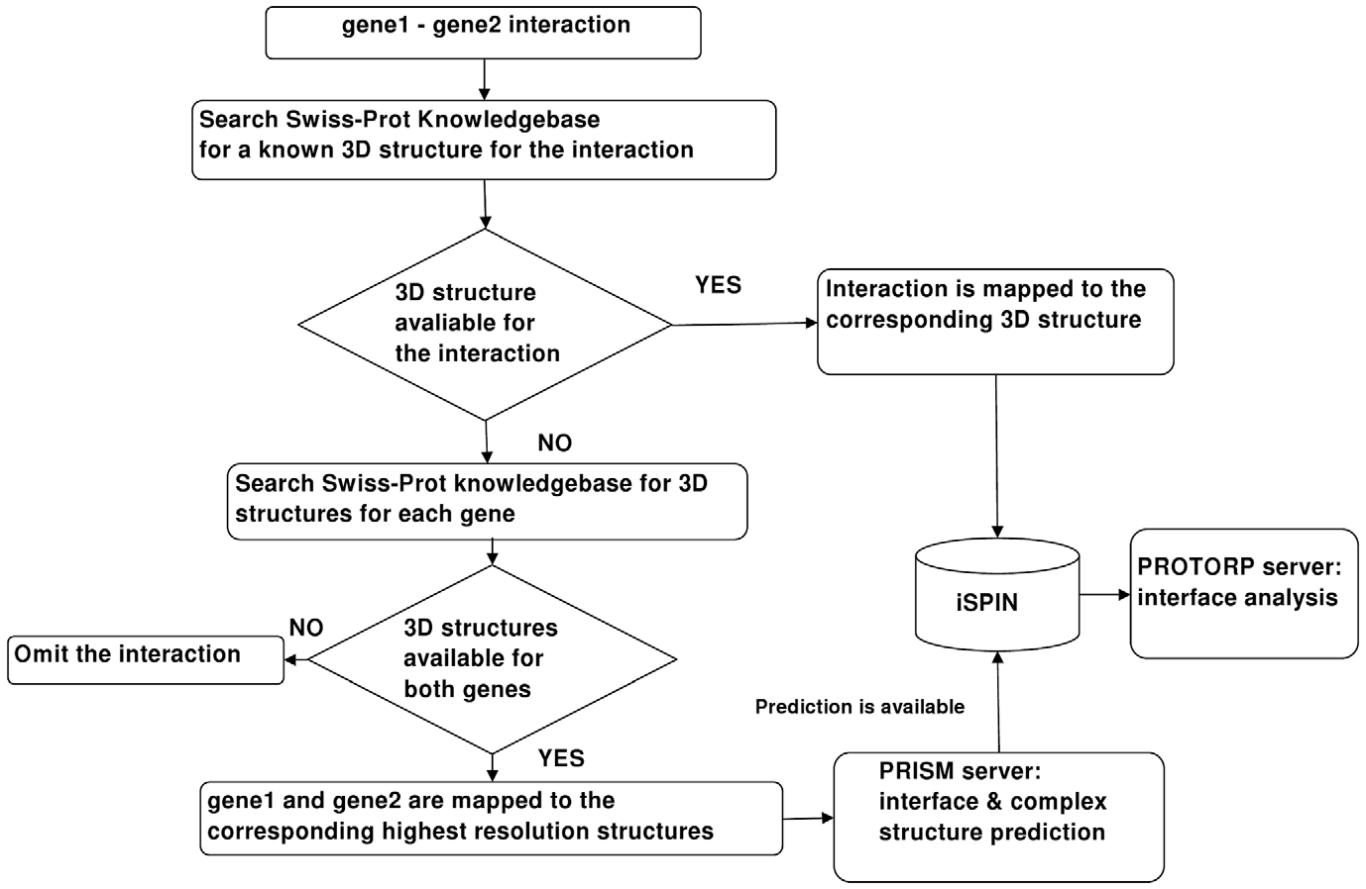
Flowchart representation of the method of mapping interactions to 3D structures and generating iSPIN. The method is applied for all the interactions in the human interactome (PIN).

We used Swiss-Prot Knowledgebase [68] to map the binary interactions to known structures. The human genes for which 3D structures are known were compiled from the Swiss-Prot Knowledgebase. For each gene-gene interaction in the human interactome, a known complex structure was searched. If a known structure was not available for the interaction, we searched for the structures of each gene and mapped each gene to the corresponding structure as a single chain. If any of the genes in the binary interaction did not have a structural representation, then that interaction was omitted. For example, in the human interactome, one of the binary interactions is TP53-MDM2 interaction. The interaction is represented by a known complex structure in PDB [28] as 1ycr. However, for the TP53-MDM4 interaction, there occurs no known complex structure. In this case, TP53 was represented by its corresponding structure with the highest resolution for which the PDB ID is 1aie_chain A. Similarly, for MDM4, the structure is 2cr8_chainA. In total, 206 interactions were mapped to known complexes. The summary of the mapping procedure is illustrated in Figure 11.

The mapped protein-protein interaction network called the “structural protein interaction network” (SPIN) consists of 1702 nodes (proteins) and 5312 edges (interactions). From 5312 interactions, 206 interactions were mapped to known 3D structures. Therefore, the interfaces of these 206 interactions were known. On the other hand, the interfaces of the remaining 5106 interactions were left for further prediction.

When the list of cancer-related proteins were searched through 1702 proteins, 466 of them were found to be encoded by cancer-related genes (cancer gene information from Futreal et al. [67] and the Memorial Sloan Kettering computational biology website CancerGenes (http://cbio.mskcc.org/CancerGenes/Select.action), the rest (1236) were taken as encoded by noncancer genes. As a result, we defined the ‘cancer structural subnetwork’ (‘cSPIN’), as the one consisting of cancer-cancer and cancer-noncancer gene interactions. Our cSPIN contains 1303 proteins and 3221 interactions. The total number of proteins and interactions for each network is summarized in Table 5.

### Definition of hubs and bottlenecks

Degree represents the number of interaction partners of a protein. Betweenness is a measure of the total number of shortest paths going through a certain node or edge in the network [69]. We defined as hubs the proteins that are in the top 20% of the degree distribution in PIN and SPIN. That corresponds to proteins with ≥9 interactions. Accordingly, we defined bottlenecks as the top 20% proteins with the highest betweenness values. (Varying the threshold from 10% to 30% had no significant impact on our results; see Text S1 for hub/non-hub interface statistics). To calculate betweenness within the network, we used NetworkX (NX) (https://networkx.lanl.gov/wiki), a Python package. Hubs were classified as hub-bottlenecks and hub-nonbottlenecks according to high betweenness or low betweenness, respectively.

### Determination of essential human genes

Goh et al (2007) [2] predicted the essentiality of a human gene using phenotype information of the corresponding mouse orthologs. A human gene was defined as “essential” if a knock-out of its mouse ortholog results in lethality. Here embryonic/prenatal lethality and postnatal lethality are considered lethal phenotypes, and the rest of the phenotypes are considered non-lethal. We obtained the human-mouse orthology and mouse phenotype data from Mouse Genome Informatics (http://www.informatics.jax.org ) on May 10, 2008. Of 1702 proteins in our SPIN, 1536 have mouse orthologs and phenotype information. According to our classification, we found 497 genes to be essential and the rest to be non-essential.

### Predicting protein-protein interfaces in SPIN

PRISM (protein interactions by structural matching) [26],[27] is a web server to predict protein-protein interactions and protein interfaces. The prediction algorithm uses structural and evolutionary similarities to find possible binary interactions between proteins, “targets,” through similar known interfaces, “templates.” Here, target proteins were the proteins in our SPIN dataset for which we wanted to predict the interaction interfaces. As template interfaces, we used the representative interfaces generated from the nonredundant data set of protein-protein interfaces [13] available at http://prism.ccbb.ku.edu.tr/interface , for which the interactions are biological according to NOXclass [57] outputs. There are 1478 template interfaces.

The PRISM prediction algorithm starts by extracting the surfaces of target proteins by invoking NACCESS [70]. Template interfaces are split into their complementary partner chains and these partners are structurally aligned with the surfaces of the target proteins. Similarity between the target surface and one partner of the template interface is measured using a scoring function based on two factors. The first is structural similarity, in which RMSD and residue match ratio between target protein and the template interface is scored. The other factor considers evolutionary similarity in which a hotspot match ratio is scored. (Critical residues at the interface which account for the majority of the binding free energy are called hotspots [71]. PRISM obtains the information on hotspots from Hotsprint [72],[73] a web server for predicting hotspots at protein interfaces.) Then, combining these scores, PRISM predicts the most possible interactions occurring between the target proteins.

### Elimination of crystal packing interfaces and interactions

After we obtained the interfaces of the proteins in our network using PRISM, non-biological interfaces, if any, should be eliminated. Interfaces having a biological score greater than 60% according to the NOXclass [57] outputs were accepted as biologically relevant. Thus, 357 interaction interfaces were predicted and most of them (80%) had biological scores greater than 80%. Also, including the known interfaces coming from 3D structures, the resulting network which includes interface information is called ‘iSPIN’. It consists of 534 proteins and 563 interactions. The subnetwork of cancer-related interactions (ciSPIN) includes 381 proteins and 375 interactions. The protein and interaction numbers are given in Table 5.

### Hub classification: Single-interface and multi-interface hubs

Kim et al. (2006) [19] classified protein hubs as singlish-interface and multi-interface hubs. The former has at most two distinct binding interfaces, whereas the latter has more than two binding interfaces. In this study, we also classified the hubs in iSPIN according to the number of distinct binding interfaces; we defined single-interface hubs as protein hubs with only one distinct binding interface and multi-interface hubs as those with more than one distinct binding interface. To distinguish overlapping interfaces from non-overlapping interfaces, we looked at the shared residue percentage of the interfaces of hub proteins. We defined shared residue percentage as the ratio of number of shared residues to the number of total interface residues. If the interface residues are shared at a percentage greater than 20%, then the corresponding interface is an overlapping one and interactions occurring through this interface are mutually exclusive. On the other hand, if the interface is not shared at all, meaning that the shared residue percentage is less than 20%, then this is a non-overlapping interface and the interaction through this interface is simultaneously possible, independent of each other.

### Interface property analysis

For interface analysis, we used PROTORP [29] which invokes NACCESS [70], SURFNET [74] and PRINCIP (SURFNET) [74] for interface accessible surface area and gap volume and planarity calculation, respectively. PROTORP calculates the amino acid composition of residues defined in the interface as a percentage value of those classified as polar, non-polar and charged as described previously by Jones and Thornton [75]. The amino acid compositions were weighted and then normalized by the interface ASA values which were calculated using NACCESS.

### Statistical tests

Mann-Whitney test (also called Wilcoxon rank sum), which is a nonparametric test that compares the distributions of two unmatched groups, was performed to compare cancer and non-cancer related interface properties. Two-tailed p values were calculated at α = 0.05.

### Classification analysis

To check whether the differences in cancer & noncancer related interface properties are significant in practice or not, Weka [39], which is a machine learning software, was used. Training set contained equal number of cancer-related (positive set) and noncancer interfaces (negative set). To equalize the number of data in the positive and negative set, a Weka filter called “Resample” which creates a stratified subsample of the given dataset, was used. “Resample” filter ensures that overall class distributions are retained within the sample. 10 runs of 10-fold cross validation were performed using four different classifier algorithms; decision stump, naïve bayes, support vector machine (SVM) and adaboostm1. Decision stump is a machine learning algorithm consisting of a single-level Decision Tree. It is mostly used as a component in boosting algorithms such as Adaboostm1. In Weka, Adaboostm1 functions as a meta-classifier which uses decision stump by weighting several iterations of it. Naïve Bayes is a simple probabilistic classifier whereas SVM is a supervised learning classifier. The statistical measures of the tests are Accuracy and Precision. Accuracy is the percentage of correctly classified instances calculated by TP+TN/(TP+TN+FN+FP). For cancer class predictions, TP is the number of correctly predicted cancer interfaces and FP is the number of non-cancer interfaces which are predicted as cancer-related. TN is the number of correctly predicted noncancer interfaces and FN is the number of cancer-related interfaces which are predicted as being non-cancer. Precision is the proportion of the instances which are correctly predicted among all predictions and calculated by TP/(TP+FP) for cancer class. For noncancer class, precision is calculated by TN/(TN+FN). Average of two precision values (for cancer and noncancer) comes out to be Precision of the tests.

### Interaction energy calculation

For the case studies, interaction energies were calculated using FoldX [53],[54]. Firstly, the complex structures were subjected to an optimization procedure using the repair function of FoldX. During this step, all side chains were moved slightly to eliminate small van der Waals' clashes. Next, AnalyzeComplex function was used to determine the interaction energy between the proteins. Throughout the FoldX calculations, the default parameters were used.

### Network topology analysis

All the parameters describing the network topology were calculated using NetworkAnalyzer, which is a Java plugin for Cytoscape [76]. Another Cytoscape plugin MCODE [44], which detects densely connected regions in protein-protein interaction networks based on a vertex weighting method by local neighborhood density, was used to find highly connected subnetworks in the network. BINGO [77], being also a Cytoscape plugin, determines which Gene Ontology terms are significantly overrepresented in subgraphs of biological networks.

## Supporting Information

Text S1 Figure S1, Figure S2, Table S1–S3
(2.02 MB DOC)
