# Supplementary material for: Human Cancer Protein-Protein Interaction Network: A Structural Perspective
Source: PLoS Comput Biol. 2009 Dec 11;5(12):e1000601. doi: 10.1371/journal.pcbi.1000601 (PMC2785480; doi:10.1371/journal.pcbi.1000601)
Supplement: Text S1 — Figure S1, Figure S2, Table S1–S3 (2.02 MB DOC) [file pcbi.1000601.s001.doc]

**Supplementary Table 1. Cancer/noncancer classification analysis and statistical test results for iSPIN interface data, iSPIN clustered data according to phenotype, molecular function or biological process using different classifier algorithms.**

In Weka results columns, first line is Accuracy value and second line is Precision value. cr stands for cancer relates interactions, ncr stands for noncancer interactions

| Group | Classification performances | | | | Cancer/Noncancer  ASA complex  ΔASA  Planarity  Gap V.I | p-value  (at α=0.05) |
| --- | --- | --- | --- | --- | --- | --- |
|  | Decision stump | Naïve Bayes | SVM | Adaboostm1 |  |  |
| All data in iSPIN  (363 cr – 186 ncr) | 0.64 0.58 | 0.67 0.64 | 0.66 0.44 | 0.65 0.60 | 2210.9 (±1476.0) / 2628.1 (±1947.5)  1009.1 (±611.77) / 1243.0 (±942.69)  2.843 (±1.285) / 3.056 (±1.233)  2.760 (±1.482) / 2.543 (±1.275) | 0.0006  6.2e-005  0.0429  0.0798 |
| iSPIN equal # of instances  (186 cr -186 ncr) | 0.54 0.57 | 0.54 0.55 | 0.54 0.54 | 0.54  0.54 | 2199.6 (±1436.5) / 2628.1 (±1947.5)  1029.7 (±732.01) / 1243.0 (±942.69)  2.954 (±1.492) / 3.056 (±1.233)  2.820 (±1.651) / 2.543 (±1.275) | 0.0058  0.0013  0.1937  0.1088 |
| iSPIN PDB – PDB interfaces  (55 cr – 55 ncr) | 0.53 0.54 | 0.55 0.55 | 0.56 0.56 | 0.56 0.56 | 1917.2 (±884.04) / 2471.3 (±1240.9)  911.41 (±489.56) / 1258.8 (±740.15)  2.723 (±1.316) / 3.298 (±1.424)  3.018 (±1.833) / 2.278 (±1.149) | 0.03  0.0089  0.0228  0.0214 |
| iSPIN predicted interfaces  (131 cr – 131 ncr) | 0.53 0.54 | 0.58 0.62 | 0.57 0.58 | 0.56 0.56 | 2186.6 (±1146.4) / 2694.0 (±2177.8)  989.81 (±347.54) / 1236.3 (±1018.3)  2.727 (±0.880) / 2.955 (±1.135)  2.584 (±1.020) / 2.654 (±1.312) | 0.0051  0.0035  0.2427  0.7967 |
| Phenotype: Leukemia  (55 cr – 55 ncr) | 0.58 0.69 | 0.60  0.63 | 0.61 0.64 | 0.61 0.63 | 1863.3 (±1207.9) / 2425.8 (±1207.9)  850.86 (±346.91) / 1147.5 (±633.31)  2.482 (±0.762) / 2.939 (±1.390)  2.862 (±1.285) / 2.423 (±0.985) | 0.0007  0.0287  0.4128  0.1125 |
| Phenotype: Breast cancer  (22 cr – 22 ncr) | 0.68 0.81 | 0.64 0.66 | 0.71 0.77 | 0.64 0.66 | 1908.5 (±623.82) / 2672.2 (±1257.3)  822.87 (±290.40) / 1343.6 (±670.89)  2.361 (±0.601) / 3.269 (±1.455)  2.306 (±1.138) / 2.239 (±0.9478) | 0.03  0.0047  0.1079  0.9159 |
| Phenotype: Colorectal cancer  (23 cr – 23 ncr) | 0.54 0.56 | 0.65 0.67 | 0.67 0.73 | 0.57 0.57 | 1923.8 (±533.87) / 2790.4 (±1352.8)  978.07 (±325.92) / 1428.5 (±771.66)  2.781 (±1.003) / 3.472 (±1.724)  2.547 (±1.748) / 2.229 (±0.9272) | 0.0167  0.04  0.2917  0.6211 |
| Molecular function: Signal Transducer Activity  (65 cr – 65 ncr) | 0.53 0.55 | 0.49 0.48 | 0.53 0.55 | 0.40  0.39 | 2226.3 (±1370.7) / 2454.7 (±1726.1)  989.50 (±423.20) / 1033.5 (±451.70)  2.886 (±1.347) / 2.805 (±1.141)  2.764 (±1.178) / 2.612 (±1.201) | 0.3814  0.6282  0.8559  0.3801 |
| Molecular function: Catalytic Activity  (84 cr – 84 ncr) | 0.58 0.68 | 0.58 0.62 | 0.58 0.62 | 0.56 0.57 | 2042.8 (±823.69) / 2758.1 (±2233.0)  963.32 (±340.09) / 1277.1 (±627.45)  2.916 (±1.446) / 3.171 (±1.320)  2.496 (±1.066) / 2.577 (±1.232) | 0.0085  0.0006  0.1078  0.6185 |
| Molecular function: Nucleic Acid Binding  (31 cr – 31 ncr) | 0.53 0.53 | 0.57 0.57 | 0.58 0.60 | 0.52 0.52 | 2229.9 (±1504.6) / 2567.5 (±870.98)  913.30 (±382.07) / 1308.0 (±508.19)  2.717 (±1.458) / 3.217 (±1.193)  2.240 (±0.959) / 1.934 (±1.031) | 0.0188  0.0016  0.0324  0.1471 |
| Molecular function: Transcription Regulator Activity  (23 cr – 23 ncr) | 0.67 0.71 | 0.61 0.61 | 0.63 0.64 | 0.63 0.63 | 2668.6 (±2031.3) / 2877.9 (±1250.0)  1106.0 (±548.00) / 1504.1 (±587.72)  2.615 (±0.897) / 3.600 (±1.486)  2.442 (±1.091) / 2.011 (±1.111) | 0.1803  0.0224  0.0182  0.1732 |

**Supplementary Table 2. The number of HB, HNB, NHB and NHNB proteins in PIN, SPIN and random network and essentiality percentages.**

|  | **PIN** | | | | **SPIN** | | | | **Random Network (RN)** | | | |
| --- | --- | --- | --- | --- | --- | --- | --- | --- | --- | --- | --- | --- |
|  | **HB** | **HNB** | **NHB** | **NHNB** | **HB** | **HNB** | **NHB** | **NHNB** | **HB** | **HNB** | **NHB** | **NHNB** |
| **Essential** | 531 | 169 | 78 | 905 | 120 | 60 | 32 | 285 | 81 | 32 | 12 | 170 |
| **Nonessential** | 1167 | 466 | 444 | 5045 | 102 | 73 | 62 | 803 | 146 | 72 | 42 | 730 |
| **Total** | 1698 | 635 | 522 | 5950 | 222 | 133 | 94 | 1088 | 227 | 104 | 54 | 900 |
| **Essentiality %** | 31 | 26 | 14 | 15 | 54 | 45 | 34 | 26 | 35 | 30 | 22 | 18 |
|  | **Cancer genes in PIN** | | | | **Cancer genes in SPIN** | | | | **Cancer genes in RN** | | | |
|  | **HB** | **HNB** | **NHB** | **NHNB** | **HB** | **HNB** | **NHB** | **NHNB** | **HB** | **HNB** | **NHB** | **NHNB** |
| **Essential** | 215 | 51 | 25 | 221 | 74 | 31 | 15 | 113 | 32 | 8 | 2 | 51 |
| **Nonessential** | 193 | 73 | 52 | 352 | 25 | 28 | 11 | 147 | 35 | 10 | 4 | 83 |
| **Total** | 408 | 124 | 77 | 573 | 99 | 59 | 26 | 260 | 67 | 18 | 6 | 134 |
| **Essentiality %** | 52 | 41 | 32 | 38 | 74 | 52 | 57 | 43 | 47 | 44 | 33 | 38 |
|  | **Hubs in PIN** | | | | **Hubs in SPIN** | | | | **Hubs in RN** | | | |
|  | **Cancer** | | **Non-cancer** | | **Cancer** | | **Non-cancer** | | **Cancer** | | **Non-cancer** | |
| **Essential** | 266 | | 434 | | 105 | | 75 | | 40 | | 73 | |
| **Nonessential** | 266 | | 1367 | | 53 | | 122 | | 45 | | 173 | |
| **Total** | 532 | | 1801 | | 158 | | 197 | | 85 | | 246 | |
| **Essentiality %** | 50 | | 24 | | 66 | | 38 | | 47 | | 30 | |

**Suplementary Table 3.** Hub-involved and nonhub-involved interface statistics with varying cutoffs for hub-definition

| **Hub definition Cutoff** | **Interface Properties** | **Hub-involved interactions** | **Nonhub-involved interactions** | **p-value** |
| --- | --- | --- | --- | --- |
| **10%**  **317 h – 232 nh** | **ASA complex** | 2171.0 (±1176.6) | 2599.9 (±2131.6) | 0.0117 |
|  | **ΔASA** | 1001.3 (±423.54) | 1207.3 (±1028.3) | 0.0158 |
|  | **Planarity** | 2.829 (±1.159) | 3.033 (±1.403) | 0.1627 |
|  | **Gap Volume Index** | 2.748 (±1.479) | 2.603 (±1.329) | 0.2137 |
| **15%**  **404 h -145 nh** | **ASA complex** | 2218.9 (±1285.6) | 2723.9 (±2383.9) | 0.0244 |
|  | **ΔASA** | 1008.7 (±1008.7) | 1310.4 (±1246.4) | 0.0056 |
|  | **Planarity** | 2.836 (±1.144) | 3.13 (±1.553) | 0.0831 |
|  | **Gap Volume Index** | 2.712 (±1.424) | 2.615 (±1.403) | 0.4073 |
| **20%**  **455 h – 94 nh** | **ASA complex** | 2230.0 (±1326.0) | 2943.9 (±2691.6) | 0.0030 |
|  | **ΔASA** | 1011.6 (±435.29) | 1459.9 (±1484.4) | 0.0004 |
|  | **Planarity** | 2.825 (±1.136) | 3.349 (±1.726) | 0.0099 |
|  | **Gap Volume Index** | 2.725 (±1.403) | 2.499 (±1.480) | 0.0580 |
| **25%**  **463 h – 86 nh** | **ASA complex** | 2225.4 (±1317.7) | 3035.5 (±2790.5) | 0.0012 |
|  | **ΔASA** | 1009.0 (±433.06) | 1515.7 (±1539.1) | < 0.0001 |
|  | **Planarity** | 2.823 (±1.132) | 3.409 (±1.774) | 0.0050 |
|  | **Gap Volume Index** | 2.729 (±1.393) | 2.458 (±1.532) | 0.0145 |
| **30%**  **492 h – 57 nh** | **ASA complex** | 2332.8 (±1695.4) | 2520.5 (±1326.2) | 0.2285 |
|  | **ΔASA** | 1072.7 (±751.15) | 1223.6 (±712.28) | 0.1107 |
|  | **Planarity** | 2.913 (±1.255) | 2.932 (±1.408) | 0.5948 |
|  | **Gap Volume Index** | 2.707 (±1.381) | 2.514 (±1.706) | 0.0266 |
| **35%**  **508 h – 41 nh** | **ASA complex** | 2353.2 (±1697.1) | 2340.1 (±1134.1) | 0.8334 |
|  | **ΔASA** | 1079.0 (±755.96) | 1204.9 (±637.86) | 0.1244 |
|  | **Planarity** | 2.913 (±1.260) | 2.934 (±1.412) | 0.7705 |
|  | **Gap Volume Index** | 2.720 (±1.409) | 2.274 (±1.474) | 0.0016 |
| **40%**  **508 h – 41 nh** | **ASA complex** | 2353.2 (±1697.1) | 2340.1 (±1134.1) | 0.8334 |
|  | **ΔASA** | 1079.0 (±755.96) | 1204.9 (±637.86) | 0.1244 |
|  | **Planarity** | 2.913 (±1.260) | 2.934 (±1.412) | 0.7705 |
|  | **Gap Volume Index** | 2.720 (±1.409) | 2.274 (±1.474) | 0.0016 |


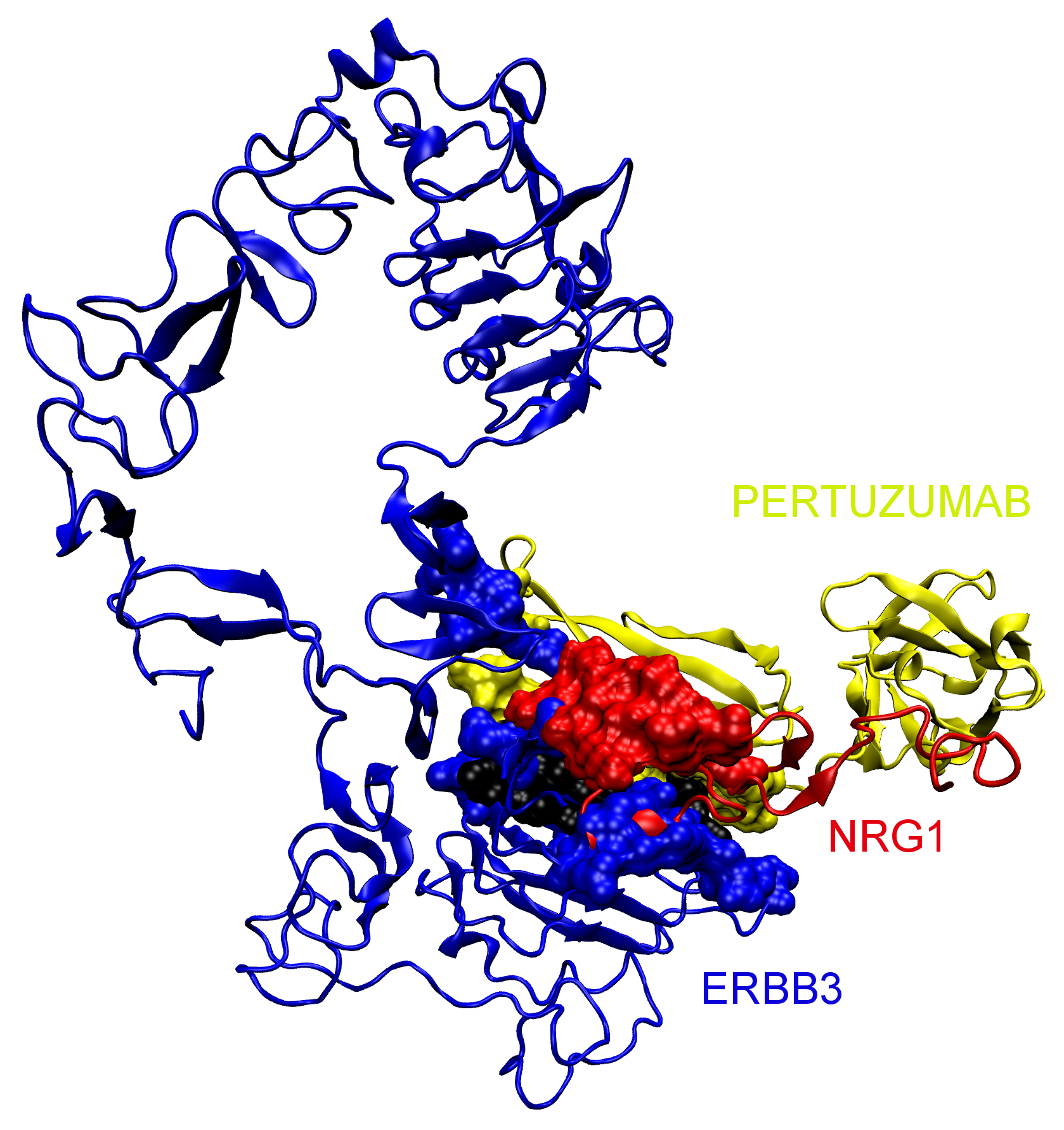


**SF1. Case study of ERBB3.**

**
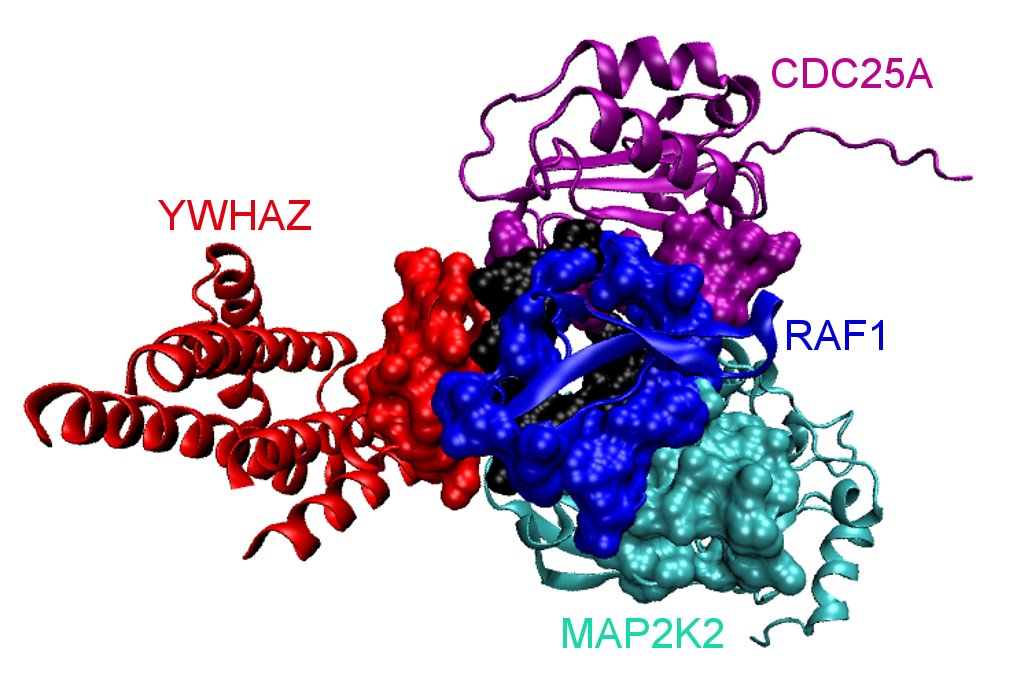
**

**SF2. Case study of RAF1.**
